# Comparative Efficacy and Safety of Different Antiplatelet Agents for Prevention of Major Cardiovascular Events and Leg Amputations in Patients with Peripheral Arterial Disease: A Systematic Review and Network Meta-Analysis

**DOI:** 10.1371/journal.pone.0135692

**Published:** 2015-08-14

**Authors:** Konstantinos Katsanos, Stavros Spiliopoulos, Prakash Saha, Athanasios Diamantopoulos, Narayan Karunanithy, Miltiadis Krokidis, Bijan Modarai, Dimitris Karnabatidis

**Affiliations:** 1 Department of Interventional Radiology, Guy's and St. Thomas' Hospitals, NHS Foundation Trust, King's Health Partners, London, United Kingdom; 2 Department of Interventional Radiology, Patras University Hospital, School of Medicine, Rion, Greece; 3 Academic Department of Surgery, Cardiovascular Division, Kings College London, BHF Centre of Research Excellence & NIHR Biomedical Research Centre at King’s Health Partners, St. Thomas' Hospital, London, United Kingdom; 4 Department of Interventional Radiology, Addenbrooke’s Hospital, Cambridge University Hospitals NHS Foundation Trust, Cambridge, United Kingdom; Maastricht University Medical Center, NETHERLANDS

## Abstract

There is a lack of consensus regarding which type of antiplatelet agent should be used in patients with peripheral arterial disease (PAD) and little is known on the advantages and disadvantages of dual antiplatelet therapy. We conducted a systematic review and network meta-analysis of available randomized controlled trials (RCT) comparing different antiplatelet drugs (Aspirin, Ticlopidine, Clopidogrel, Ticagrelor, Cilostazol, Picotamide and Vorapaxar as monotherapies or in combination with aspirin) in PAD patients (PROSPERO public database; CRD42014010299).We collated evidence from previous relevant meta-analyses and searched online databases. Primary efficacy endpoints were: (1) the composite rate of major adverse cardiovascular events (MACE; including vascular deaths, non-fatal myocardial infarction and non-fatal stroke), and (2) the rate of major leg amputations. The primary safety endpoint was the rate of severe bleeding events. Bayesian models were employed for multiple treatment comparisons and risk-stratified hierarchies of comparative efficacy were produced to aid medical decision making. Number-Needed-to-Treat (NNT) and Number-Needed-to-Harm (NNH) are reported in case of significant results. We analyzed 49 RCTs comprising 34,518 patients with 88,358 person-years of follow-up with placebo as reference treatment. Aspirin, Cilostazol, Vorapaxar and Picotamide were ineffective in reducing MACE. A significant MACE reduction was noted with Ticagrelor plus aspirin (RR: 0.67; 95%CrI: 0.46–0.96, NNT = 66), Clopidogrel (RR: 0.72; 95%CrI: 0.58–0.91, NNT = 80), Ticlopidine (RR: 0.75; 95%CrI: 0.58–0.96, NNT = 87), and Clopidogrel plus aspirin (RR: 0.78; 95%CrI: 0.61–0.99, NNT = 98). Dual antiplatelet therapy with Clopidogrel plus aspirin significantly reduced major amputations following leg revascularization (RR: 0.68; 95%CrI: 0.46–0.99 compared to aspirin, NNT = 94). The risk of severe bleeding was significantly higher with Ticlopidine (RR: 5.03; 95%CrI: 1.23–39.6, NNH = 25), Vorapaxar (RR: 1.80; 95%CrI: 1.22–2.69, NNH = 130), and Clopidogrel plus aspirin (RR: 1.48; 95%CrI: 1.05–2.10, NNH = 215). Clopidogrel monotherapy showed the most favourable benefit-harm profile (79% cumulative rank probability best and 77% cumulative rank probability safest). In conclusion, Clopidogrel should be the indicated antiplatelet agent in PAD patients. Dual antiplatelet therapy with aspirin and Clopidogrel can reduce the rate of major leg amputations following revascularization, but carries a slightly higher risk of severe bleeding.

## Introduction

Peripheral arterial disease (PAD) affects between 10–20% of the western population and has become a global problem.[1] Symptoms range from intermittent claudication to tissue loss and PAD patients also have a six fold increased risk of cardiovascular related death.[2, 3] Risk factor modification including cessation of smoking, control of diabetes, diet and exercise is recommended. In addition, these patients are also advised to receive antiplatelet therapy to prevent major adverse cardiovascular events (MACE) including myocardial infarction (MI), stroke and death.[4–8] Evidence shows that single antiplatelet therapy is associated with a 25% odds reduction in MACE in a range of high-risk patients with cardiovascular diseases.[9] This includes patients with an acute or previous MI, acute or previous ischemic stroke, stable or unstable angina, and atrial fibrillation.[9] Hence, patients with symptomatic PAD usually receive single antiplatelet therapy with daily aspirin. There is, however, no consensus regarding, which specific antiplatelet should be used; the advantages and disadvantages of dual antiplatelet therapy; how best to treat the PAD population, which represents a sub-group of cardiovascular patients with risks to the heart, brain and lower limbs; and efficacy of antiplatelet drugs in preventing major leg amputations.

Indeed, a recent meta-analysis investigating the efficacy of aspirin specifically in the PAD population has suggested that Aspirin reduces the risk of non-fatal stroke but is otherwise ineffective for prevention of all-cause or cardiovascular mortality [10], while the use of dual antiplatelet treatment for PAD remains empirical depending on local practice, cardiovascular comorbidities, severity of leg symptoms, anatomical extent of the disease and treatment. Alternative antiplatelet medications including Adenosine Diphosphate (ADP) receptor antagonists (e.g. Ticlopidine, Clopidogrel and Ticagrelor)[11], phosphodiesterase inhibitors (Cilostazol),[12] thromboxane blockers (Picotamide)[13] and a novel protease-activated receptor-1 antagonist (Vorapaxar)[14] have all been proposed for prophylactic treatment in PAD patients but no trial has been able to compare the effectiveness of these medications together.

Network meta-analysis (NMA) can evaluate the comparative efficacy of a range of treatments that have or have not been compared directly against each other, provided all therapies under investigation are linked to a common chain or network of evidence.[15, 16] In this study, we conduct a systematic review and network meta-analysis to better inform health policy and medical decision making for the treatment of patients with PAD. Within a Bayesian framework, we synthesized all available randomized controlled trials (RCT) investigating the efficacy of different antiplatelet drugs (Aspirin, Ticlopidine, Clopidogrel, Ticagrelor, Cilostazol, Picotamide or Vorapaxar as monotherapies and/or in combination with Aspirin) in the prevention of MACE and leg amputations in patients with peripheral arterial disease.

## Materials and Methods

### Search and selection

This systematic review has been registered in the PROSPERO public database (CRD42014010299; http://www.crd.york.ac.uk/PROSPERO). We first identified and collated a list of randomized trials evaluating different antiplatelet medications in the PAD population from previous relevant meta-analyses.[9, 10, 17, 18] Further electronic searches of PubMed, EMBASE, AMED, Scopus, CENTRAL; FDA, EMA, and MHRA archives, the DARE and PROSPERO databases of meta-analyses, and online material were performed (last updated May 2014). The trial selection process complied with the Preferred Reporting Items for Systematic reviews and Meta-Analyses (PRISMA) statement. [19] We searched for RCTs comparing any of the aforementioned antiplatelet agents versus placebo or with each other, for prevention of MACE and/or leg amputations in the PAD population. RCTs were evaluated for inclusion in the network meta-analysis with a structured question checklist (pp 2–12; S1 Appendix).

### Endpoints and abstraction

Evaluation of the quality of RCTs was performed using the Jadad 5-point instrument for assessing risk of bias in randomized controlled trials.[20] The same tool has been used in previous systematic reviews evaluating the efficacy of aspirin.[10] An intention-to-treat principle was followed for the analysis of endpoints. There were two primary efficacy endpoints and one primary safety endpoint. The first primary efficacy endpoint was a composite endpoint of MACE consisting of all deaths from vascular causes, numbers of non-fatal MI and numbers of non-fatal stroke. The other primary efficacy endpoint was the rate of major leg amputations, which included any amputation above the ankle. This was analyzed independently as the majority of the trials did not uniformly report them. Secondary efficacy endpoints were the specific components from the primary composite endpoint (deaths from vascular causes, numbers of non-fatal MI and numbers of non-fatal stroke) analyzed individually. The primary safety endpoint was the rate of severe bleeding as reported by each study.

Separate network nodes were assigned to each individual treatment, with monotherapies treated separately from combination therapies. The only exception was dipyridamole which was pooled together with aspirin to maintain consistency with the design and results of older meta-analyses.[9, 10] We also constructed a condensed secondary network to investigate the comparative efficacy and safety of different drug classes. All authors had unrestricted access to the datasets; the lead author performed all statistical analyses and has final overall responsibility for the submitted version of the manuscript (study guarantor). There was no funding source for this study.

### Statistical methods

First, direct pairwise meta-analyses of head-to-head comparisons were performed with standard frequentist methods. Second, both the extended and condensed networks of RCTs were synthesized with Bayesian inference (WinBUGS 1.4.3, MRC Biostatistics Unit, Cambridge, United Kingdom). Bayesian hierarchical modeling of the present network meta-analysis complied with the guidelines of the National Institute for Health and Excellence Decision Support Units (NICEDSU). [21] All endpoints were recorded as counts of events per 100 person-years (Event Rates) to account for the variable follow-up period of different RCTs, and were analysed using a Bayesian fixed effects Poisson model to calculate pairwise Rate Ratios (RR) between different treatments (WinBUGS code example S1 Appendix page 53).

We constructed rankograms of cumulative rank probabilities of how each treatment ranks against each other in terms of being the 1st, 2nd, 3rd, etc best treatment option. A hierarchy of the efficacy and safety of the various antiplatelet treatments based on their cumulative rank probabilities and the Surface Area Under the Cumulative Rankograms (SUCRA, %) was then calculated as proposed by Salanti et al. [22] The *Cochran’s Q (chi* ^*2*^ *)* and the *I* ^*2*^ statistical tests were used to analyse heterogeneity. Small study effects and publication bias were evaluated by visual inspection of funnel plots of direct comparisons. In addition, extensive consistency, sensitivity and metaregression analyses were performed to test the validity and robustness of the results as outlined in the Full methods section of the S1 Appendix (pp 2–12).

### WinBUGS modelling

WinBUGS code was written and adapted according to recommendations of the NICE Decision Support Units (http://www.nicedsu.org.uk/) [21] We chose a fixed effects model because a Bayesian random effects model either converged poorly because of overparameterization or underpowered the analysis so that it did not identify significant differences in cases of treatments that were significantly different in both the fixed and random effects frequentist models (S1 Appendix pp 6–8).

Because of conceptual differences in study designs and differences in baseline demographics, the observed baseline risk of cardiovascular events varied widely between the reference treatment arms. Baseline risk is a proxy for unmeasured but important patient-level characteristics that may relate to significant clinical heterogeneity. Hence, we extended our analysis to a meta-regression model on trial-specific baseline risk of the control arms to account for the uncertainty and clinical heterogeneity introduced by the variable baseline characteristics of PAD cohorts as described.[23, 24]

To better inform decision-making and aid interpretation of the results from a clinical viewpoint, a hierarchical risk stratification analysis of the pooled treatment effects was carried out. The meta-regression coefficients of baseline risk analysis were combined with the uncertainty surrounding the posterior medians of the rate ratios of events for each treatment in order to calculate the level of risk where each treatment is projected to reach statistical significance. Minimally informative priors for effect sizes and precisions were used for all Bayesian calculations to avoid bias. Three Markov chains were compiled and ran. Convergence was confirmed with the Brooks–Gelman–Rubin diagnostic tool [25] and by inspection of history plots of monitored nodes. An initial burn-in simulation of 50,000 iterations was discarded and inference of final summary statistics was based on simulation of an additional 100,000 iterations.

## Results

### Network of evidence

**Fig 1:**
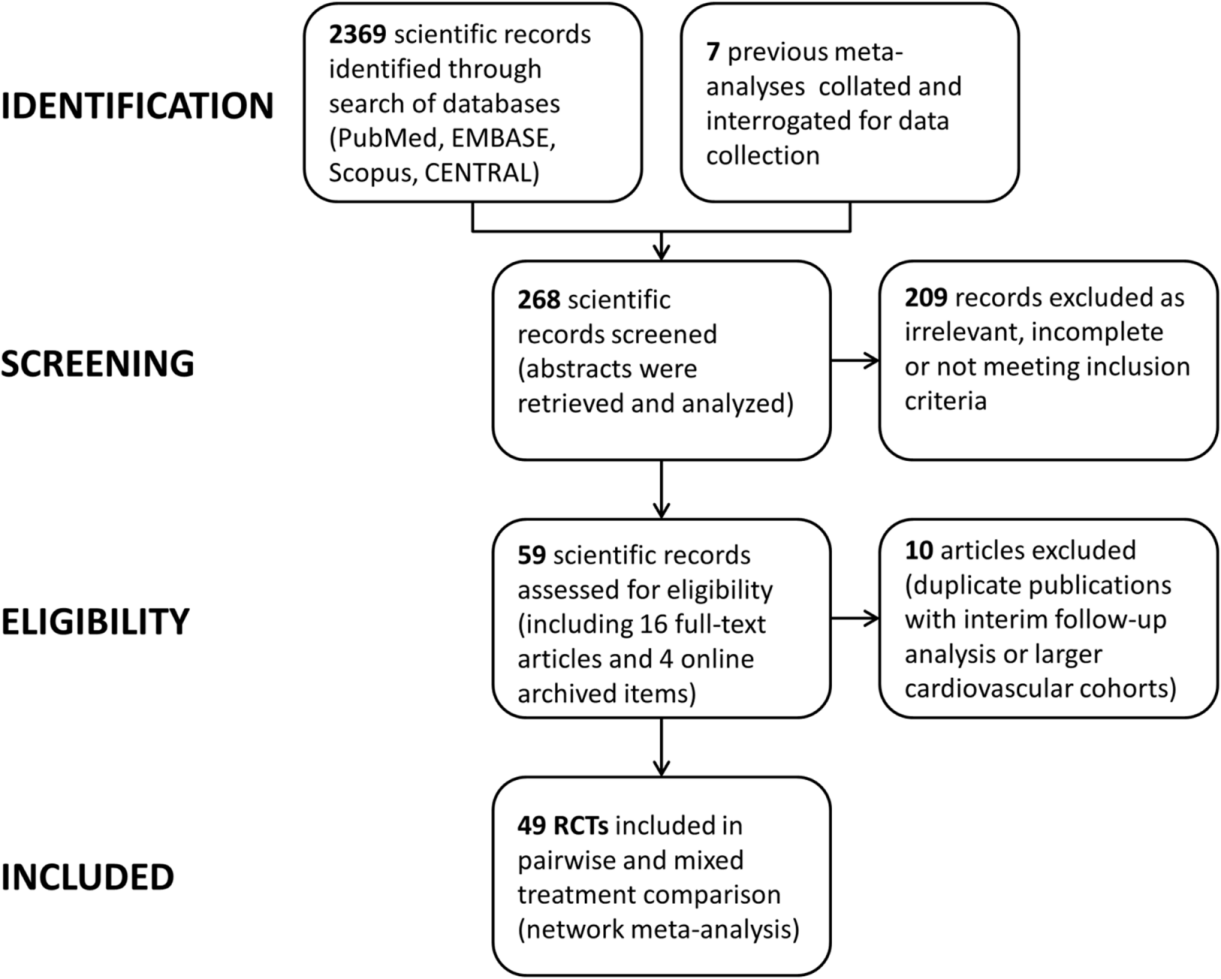
PRISMA flowchart. Trial selection process according to the PRISMA statement.

**Fig 2:**
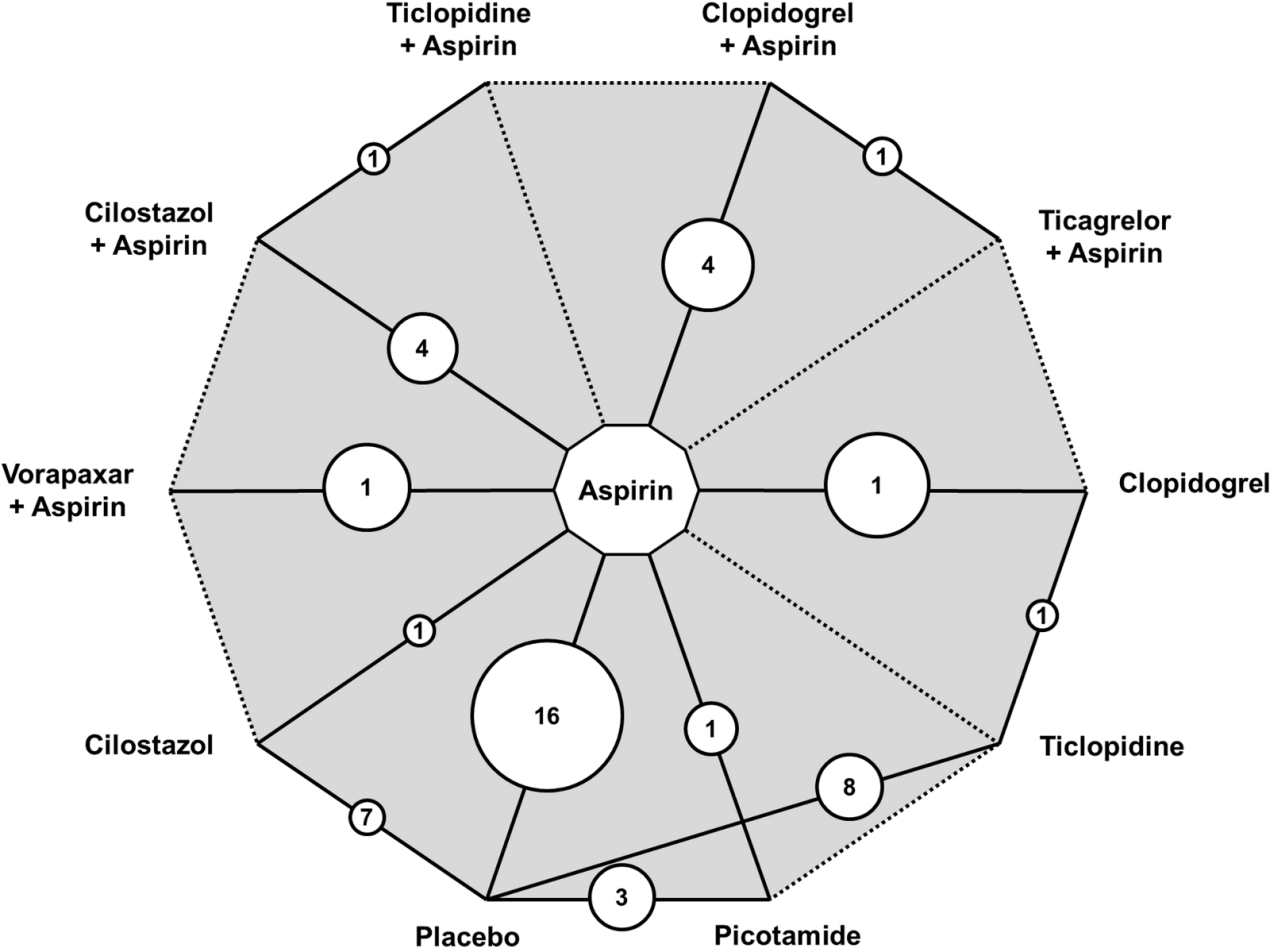
Network of evidence. Straight lines denote direct head-to-head comparisons and dotted lines denote indirect comparisons where direct comparison data is missing. Numbers refer to the number of RCTs with direct comparisons available for each link and the size of circles is proportional to the pooled sample size (person-years) available for each direct comparison.

**Fig 3:**
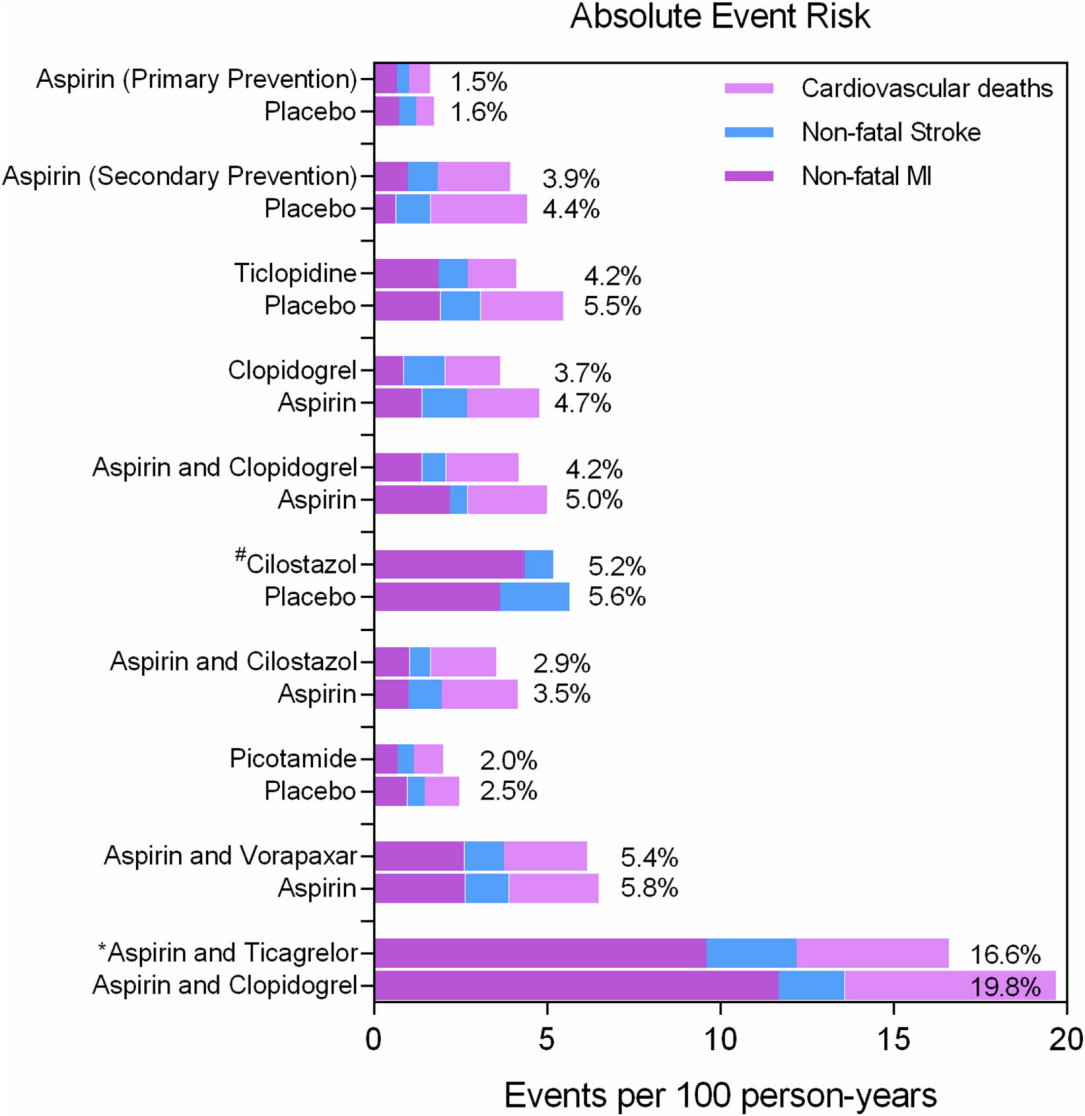
Observed risk of events in control and active arms of included RCTs. Aggregate risks of events are reported for each node and expressed as percent person-years. ^#^ In case of the Cilostazol versus placebo trials the numbers of cardiovascular deaths were not available separately, but fatal and no-fatal MIs and strokes were reported. * The PLATO trial included patients with acute coronary syndrome (ACS) at baseline.

Following the PRISMA selection process (Fig 1), the title and abstract of 2,369 scientific records were screened for potential inclusion in the network meta-analysis. From those, 49 RCTs published between 1975 and 2014 comprising 34,518 patients with 88,358 person-years of follow-up were included and analyzed. Median follow-up was 1 year on a trial basis (interquartile range, 0.5–2 years). The primary network of evidence is shown in Fig 2. The condensed secondary network and the characteristics and quality assessment of included trials are outlined in the S1 Appendix (pp 14–25). The network was well connected and nearly all trials investigated a single agent versus placebo or aspirin until 2005. Since 2005, study designs involved a combination of aspirin and another antiplatelet. Direct evidence was available for 13 comparisons and nearly half of them (n = 7) were informed from a single RCT. Study quality could not be assessed in 6 trials and another 7 were of low to medium quality (Jadad score 0–3). The majority of included studies (n = 36), however, were high quality RCTs (Jadad score 4–5). Two trials investigated aspirin versus placebo for primary prevention of cardiovascular events in asymptomatic patients with an abnormal ankle-brachial index (ABI).[26, 27] Hence, we also did separate sensitivity analyses by excluding the 2 primary prevention trials and the low-quality trials (Jadad score<3) in particular. Intermittent claudication was a protocol defined criterion in many of the trials (n = 21) and variable rates of peripheral revascularization (62–100%) were reported in most of the high-volume studies (n = 22; 21,858 participants). The observed aggregate risk of cardiovascular events varied from 1.6% in the primary prevention trials [26, 27] to 19.8% in the PLATO PAD report (acute coronary syndrome at baseline) (Fig 3).[28, 29]. A detailed checklist of preferred reporting items according to the PRISMA statement may be found in S2 Appendix.

### Primary composite endpoint

**Fig 4:**
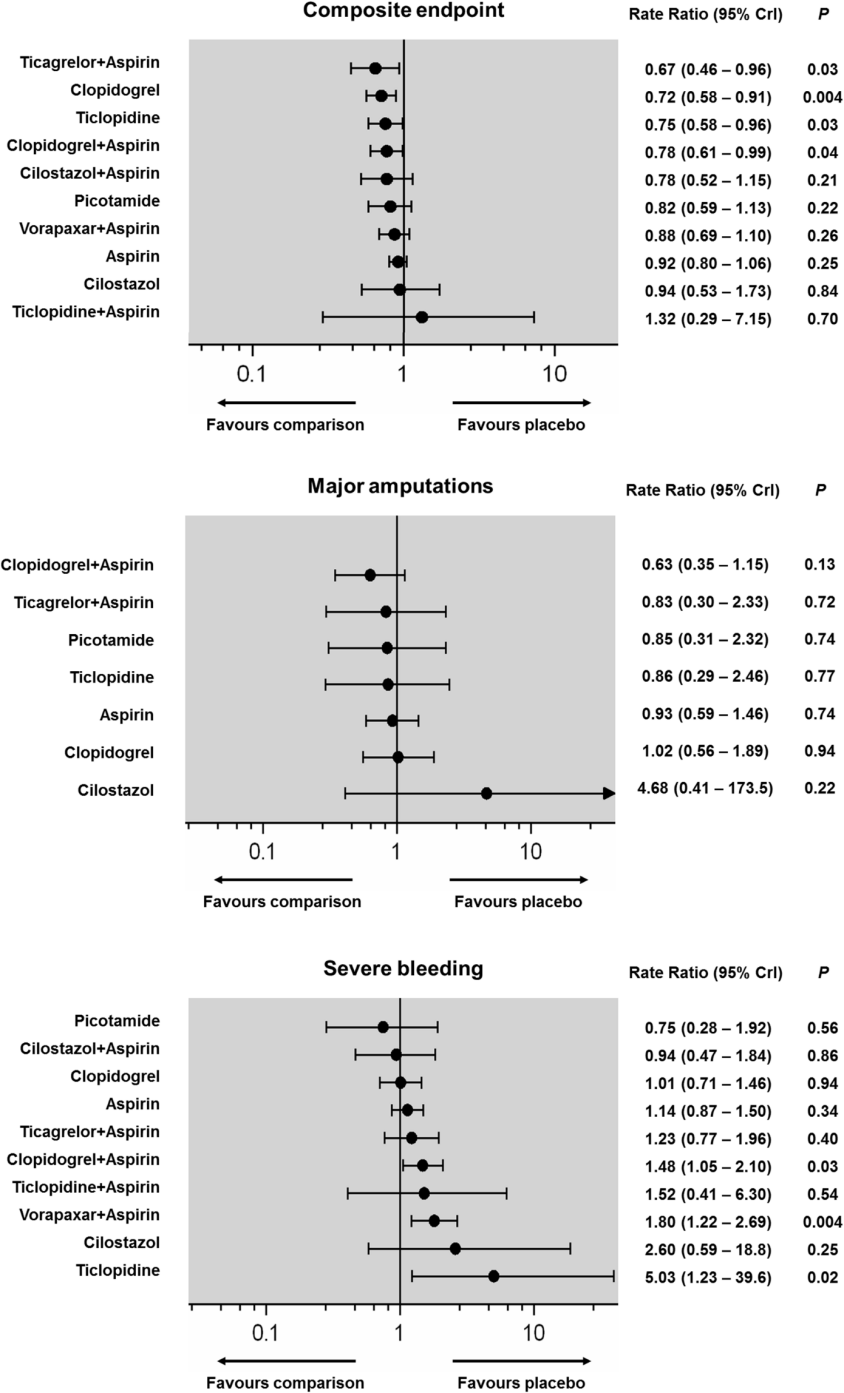
Pooled estimates for the MACE composite endpoint, major leg amputations and severe bleeding (Fixed effects forest plot). Antiplatelets are reported in order of efficacy or safety ranking. Black circles denote the posterior median and the black lines denote the associated 95% CrI. Numbers represent rate ratios (RR) and 95% CrIs. P values were approximated by the two-tailed posterior probabilities.

**Fig 5:**
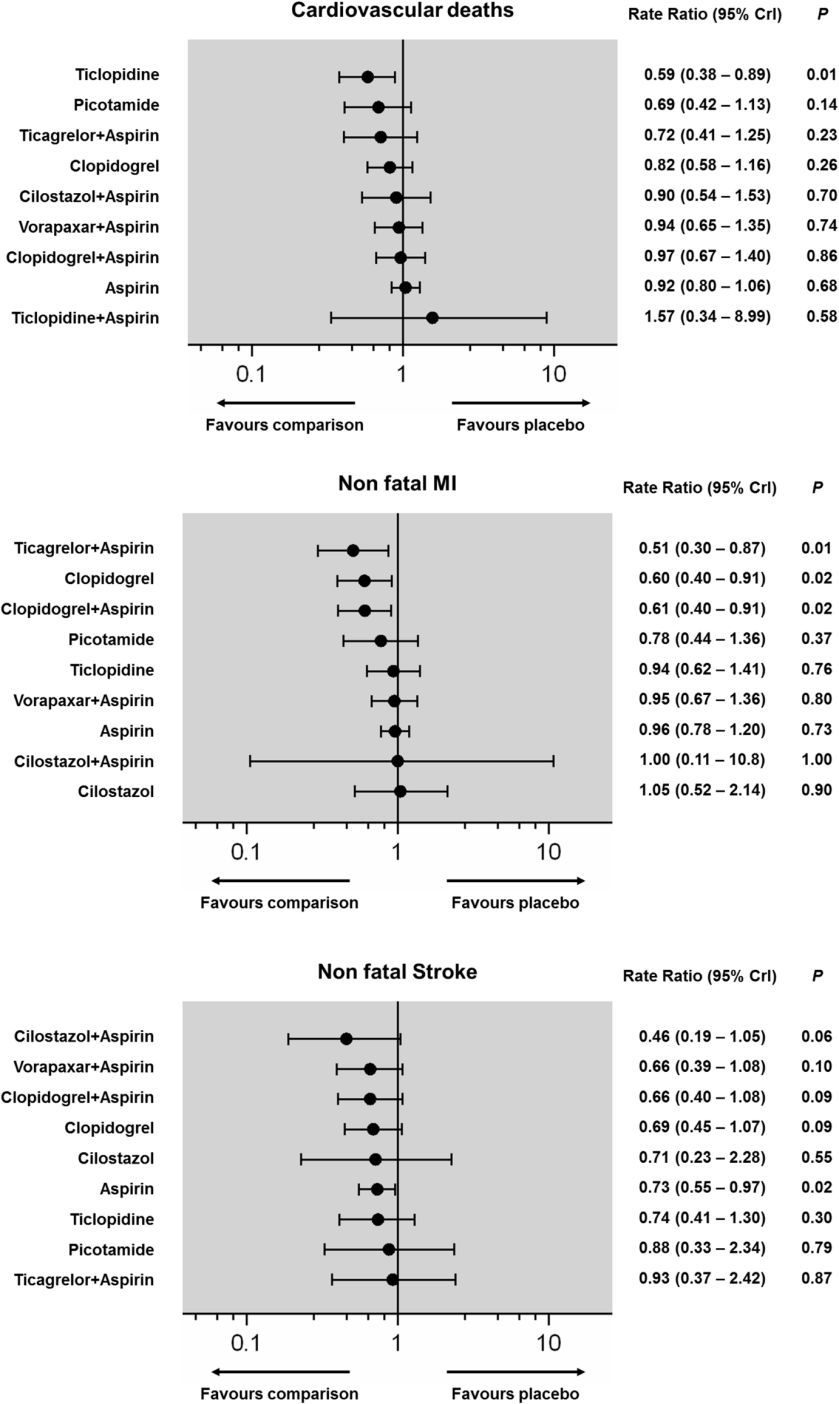
Pooled estimates for cardiovascular deaths, non-fatal MI and non-fatal stroke (Fixed effects forest plot). Antiplatelets are reported in order of efficacy ranking. Black circles denote the posterior median and the black lines denote the associated 95% CrI. Numbers represent rate ratios (RR) and 95% CrIs. P values were approximated by the two-tailed posterior probabilities.

**Fig 6:**
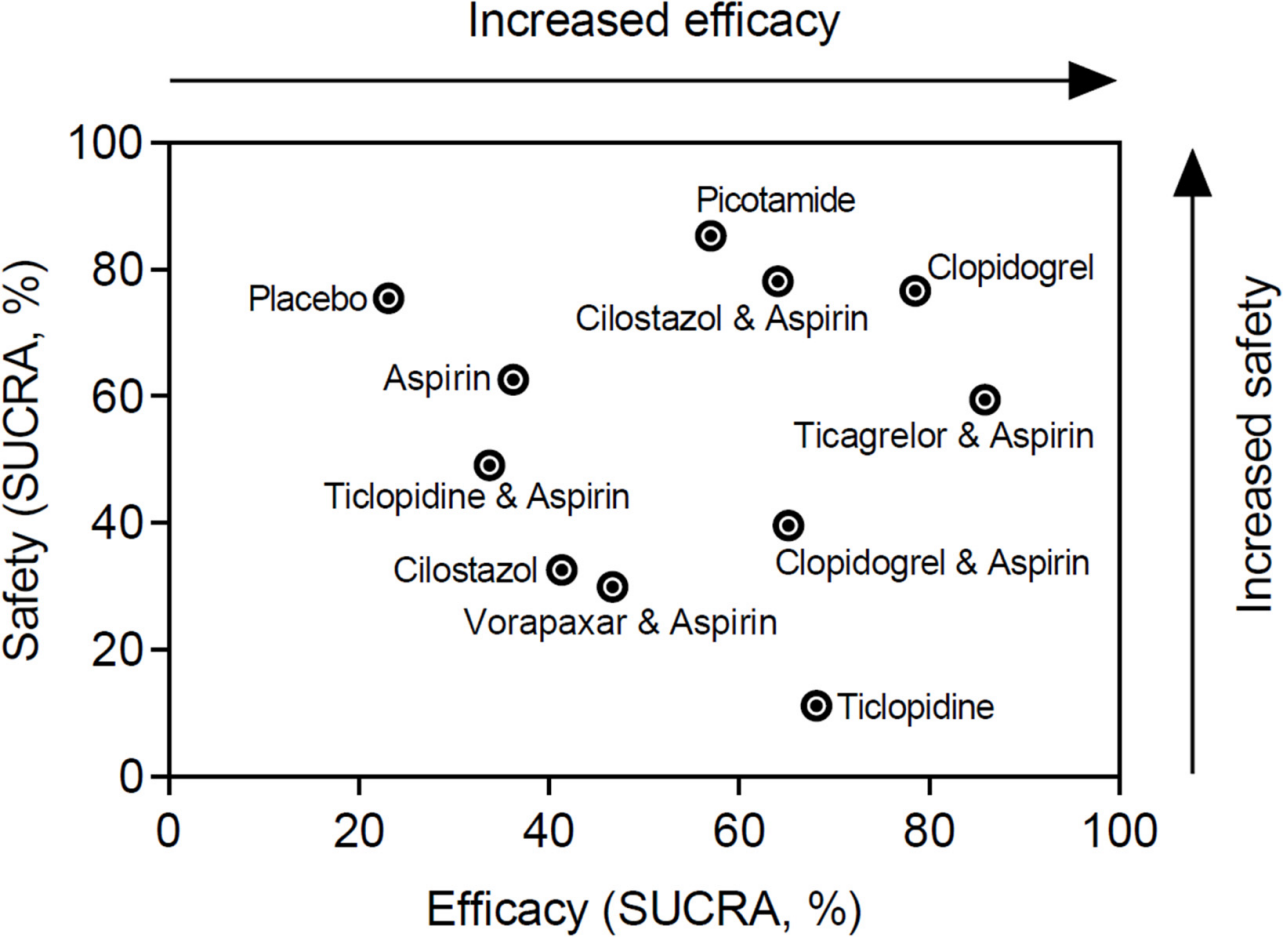
Benefit-harm profile of different antiplatelet agents. Two-dimensional ranking of antiplatelet agents according to safety (y axis) and efficacy (x axis) based on the cumulative rank probabilities (SUCRA; %). The top right corner denotes therapies with the most favourable benefit-harm profile (safe and effective).

**Fig 7:**
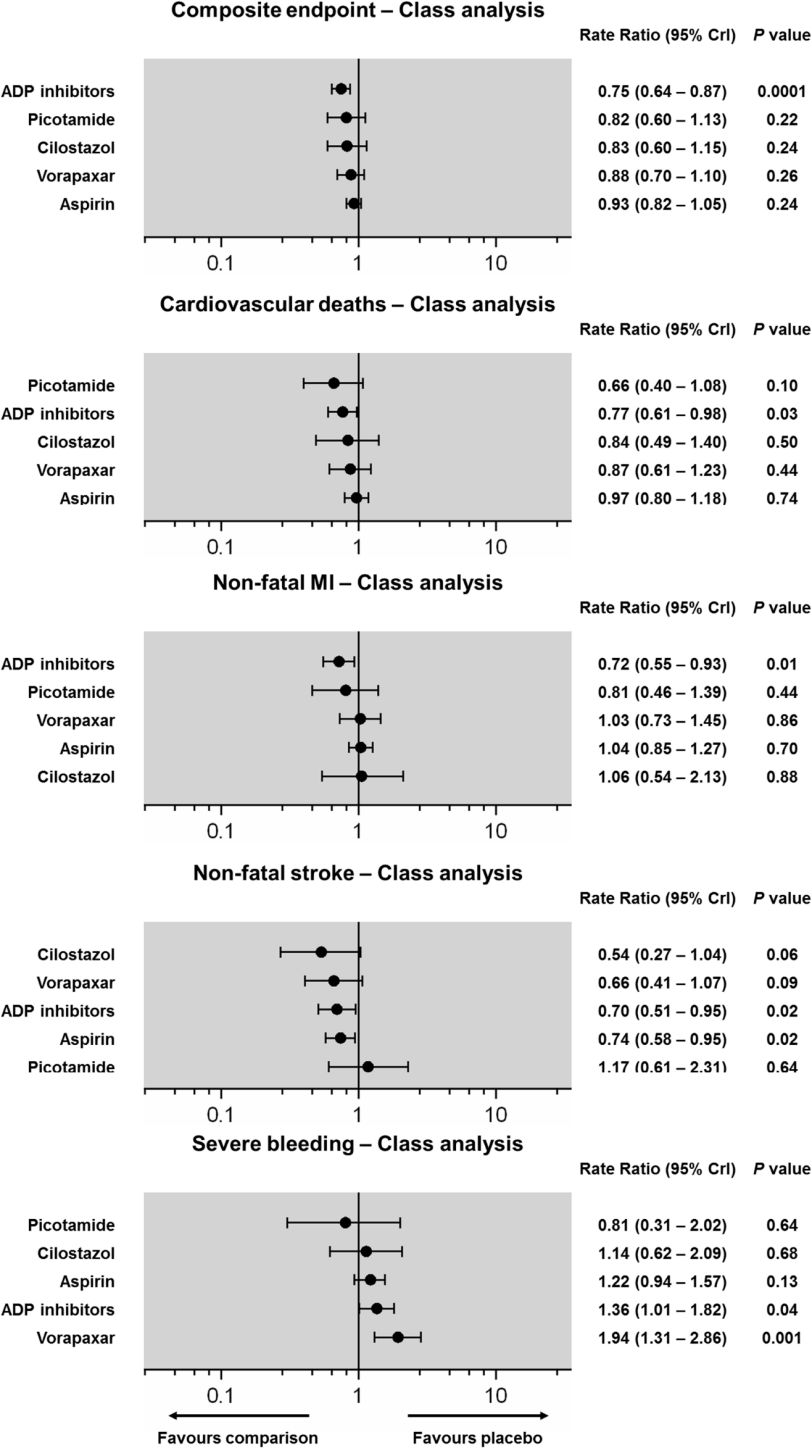
Class-level network fixed effects forest plots. Classes of antiplatelet agents are reported in order of efficacy or safety ranking. Black circles denote the posterior median and the black lines denote the associated 95% CrI. Numbers represent rate ratios (RR) and 95% CrIs. P values were approximated by the two-tailed posterior probabilities.

**Fig 8:**
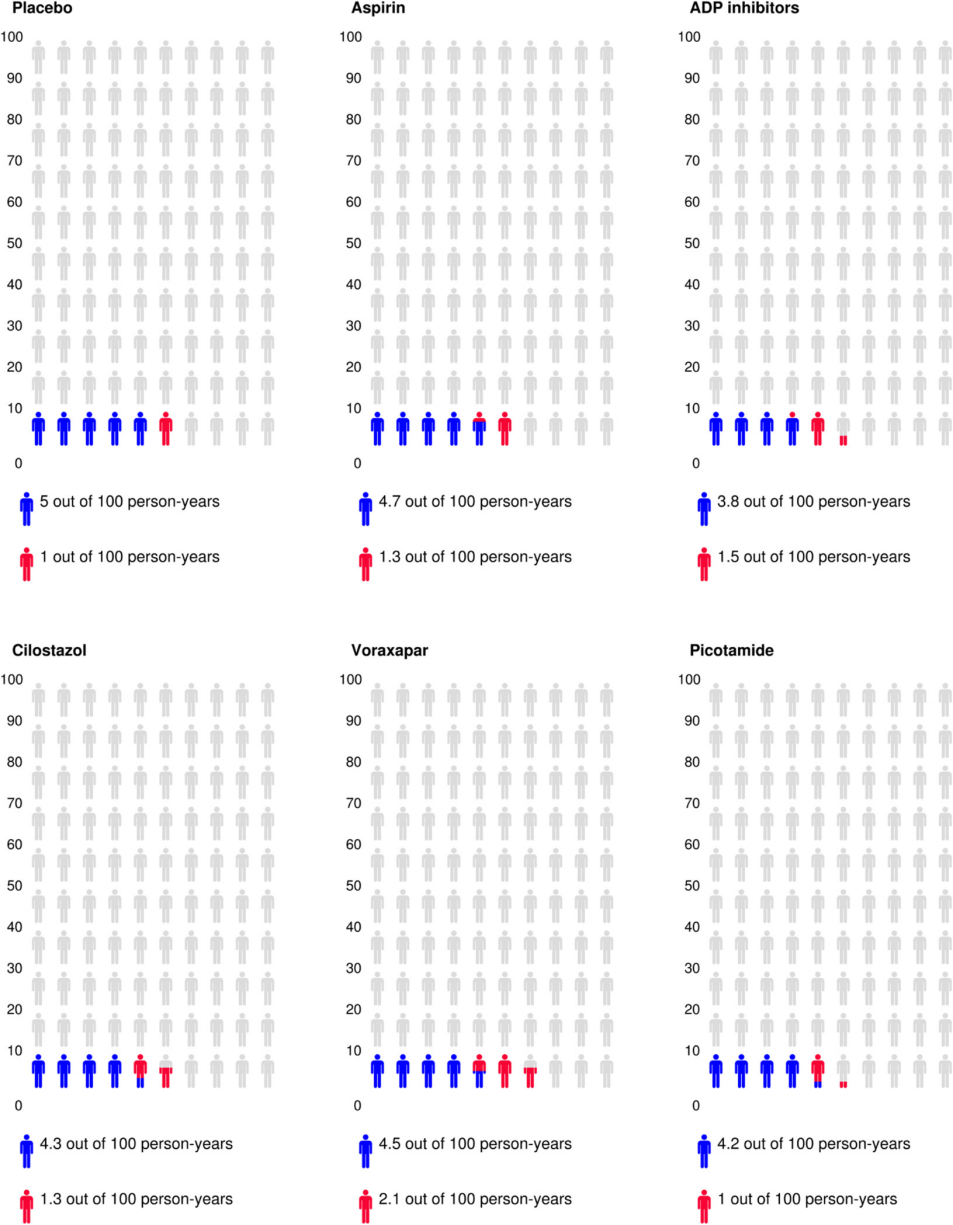
Icon arrays of absolute event rates. Icon arrays showing composite absolute event rates of MACE (blue) and severe bleeding (red) for different classes of antiplatelet agents in PAD patients. Results based on Bayesian fixed effects modelling of the secondary condensed class-level network of evidence.

All 49 RCTs contributed evidence for 13 head-to-head comparisons (n = 4 with zero total events). Fixed effects inverse variance weighted models of all direct pairwise comparisons are provided in detail in the S1 Appendix (pp 26–42). There was minimal inter-trial heterogeneity (subgroup and overall I^2^ = 0%). The hierarchies of efficacy and safety on the basis of the posterior RR (95% CrI) for all primary and secondary outcomes are shown in Figs 4 and 5. Hierarchies of different antiplatelets on the basis of cumulative rank probabilities (SUCRA, %) are shown in the S1 Appendix (p 43). Aspirin, Cilostazol, Vorapaxar and Picotamide were largely ineffective. Ticagrelor plus aspirin, Clopidogrel, Ticlopidine, and Clopidogrel plus aspirin achieved a significant 22% to 33% reduction of the composite rate of MACE (NNT range, 66–98). A significant MACE reduction was noted with Ticagrelor plus aspirin (RR: 0.67; 95%CrI: 0.46–0.96, NNT = 66), Clopidogrel (RR: 0.72; 95%CrI: 0.58–0.91, NNT = 80), Ticlopidine (RR: 0.75; 95%CrI: 0.58–0.96, NNT = 87), and Clopidogrel plus aspirin (RR: 0.78; 95%CrI: 0.61–0.99, NNT = 98). On the basis of SUCRA cumulative rank probabilities, Clopidogrel monotherapy was associated with the most favorable harm-benefit profile, as it combined a strong effect size (79% best) without being associated with an increased risk of severe bleeding (77% safest) (Fig 6). Results of the class level analysis (Fig 7) were in line with the multiple-treatment synthesis of the individual agents and only the group of ADP antagonists achieved a highly significant 25% risk reduction of the composite endpoint (RR: 0.75; 95% CrI: 0.64–0.87, NNT = 87). Fig 8 shows the icon arrays portraying the absolute events rates in terms of harm and benefit for the different antiplatelet classes analysed.

### Composite endpoint components

Fixed and random effects models of all available direct pairwise comparisons are provided in detail in the S1 Appendix (pp 26–42) and there was minimal heterogeneity (Cardiovascular deaths: I^2^ = 0%, non-fatal MI: I^2^ = 0%, non-fatal stroke: I^2^ = 0–37%, overall I^2^ = 0% in all cases). ADP antagonists were associated with a significant reduction of cardiovascular deaths (RR: 0.77; 95% CrI: 0.61–0.98, NNT = 246), and of these Ticlopidine monotherapy was the only single antiplatelet associated with a significant reduction of cardiovascular death (RR: 0.59; 95% CrI: 0.38–0.89, NNT = 140) (Figs 5 and 7). Event rates of non-fatal MI were significantly reduced by ADP antagonists (RR: 0.72; 95% CrI: 0.55–0.93, NNT = 177), and on an individual basis Ticagrelor plus Aspirin (RR: 0.51; 95%CrI: 0.30–0.87), Clopidogrel (RR: 0.60; 95%CrI: 0.40–0.91), and Clopidogrel plus Aspirin (RR: 0.61; 95%CrI: 0.40–0.91) were effective (NNT range, 100–126) (Figs 5 and 7). Aspirin had the strongest prophylactic effect against non-fatal stroke compared with placebo (RR: 0.73; 95% CrI: 0.55–0.97, NNT = 292) and at a class level, both ADP antagonists (RR: 0.70; 95% CrI: 0.51–0.95, NNT = 268) and aspirin (RR: 0.74; 95% CrI: 0.58–0.95, NNT = 313) offered a significant protection against stroke (Figs 5 and 7).

### Major leg amputations

Eleven trials included in this analysis contributed evidence about major leg amputations with 8 head-to-head comparisons (S1 Appendix pp 38–39; I^2^ = 0%). The CAPRIE[30] and PLATO[29] trials reported amputation rates on the whole cohort of patients. The network meta-analysis of leg amputations therefore included 47,670 patients with 81,259 person-years of follow-up, though the majority of comparisons were informed from a single RCT (forest plot in S1 Appendix pp 37). Dual antiplatelet therapy with Clopidogrel plus aspirin ranked highest (RR: 0.63, 95% CrI: 0.35–1.15 indirect comparison to placebo) and it was the only treatment associated with a significant reduction of major amputations following leg revascularization (RR: 0.68, 95% CrI: 0.46–0.99 direct comparison with aspirin; NNT = 94; Fig 4). The latter comparison was informed from 3 RCTs including both surgical (CASPAR trial [31]) and endovascular revascularizations (MIRROR [32] and CHARISMA trials [33, 34]).

### Primary safety endpoint

Forty-two of the 49 included RCTs reported data on major/severe bleeding (n = 10 with zero total events; I^2^ = 0–48%, overall I^2^ = 12%). Severe bleeding was significantly increased with Ticlopidine (RR: 5.03; 95%CrI: 1.23–39.6, NNH = 25), Vorapaxar (RR: 1.80; 95%CrI: 1.22–2.69, NNT = 130), and Clopidogrel plus aspirin (RR: 1.48; 95%CrI: 1.05–2.10, NNH = 215; Fig 4). The class analysis confirmed a significantly higher risk of bleeding with the use of ADP antagonists (RR: 1.36, NNH = 282). Overall, as shown in the benefit/harm icon arrays (Fig 8), ADP antagonists had the highest effect size (absolute composite rate of 3.8 events compared to 5.0 events per 100 person-years in the placebo group), but were associated with the second highest risk of severe bleeding (absolute bleeding rate of 1.5 events compared to 1.0 events per 100 person-years in the placebo group). The highest absolute rate of severe bleeding was noted with Vorapaxar plus aspirin (2.0 events / 100 person-years).

### Baseline risk meta-regression

**Fig 9:**
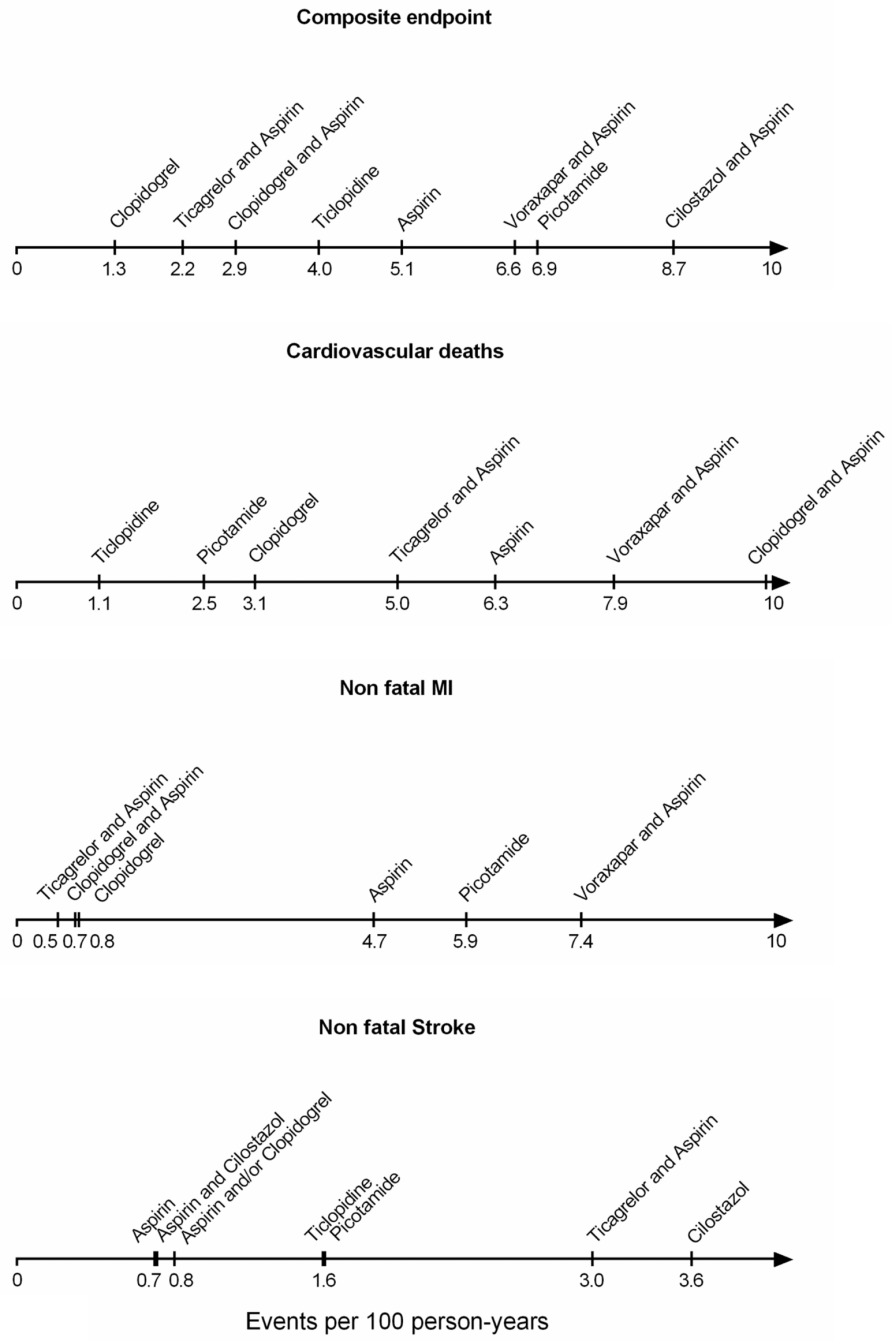
Hierarchical risk stratification analysis. Meta-regression was employed to address baseline risk variability and produce risk stratified hierarchies of antiplatelet comparative efficacy. Coefficients of baseline risk meta-regression analysis were combined with the uncertainty surrounding the posterior medians of the rate ratios of events for each treatment to calculate the level of risk where each treatment is projected to reach statistical significance (97.5% CrI of the posterior median crosses unity). Numbers refer to percent person-years of baseline risk of events (low risk: <1%, intermediate risk: 1–2%, high risk: >2%).

There was a strong interaction between baseline risk and the calculated effect sizes for the composite endpoint and the secondary endpoints of cardiovascular death, non-fatal MI and non-fatal stroke (Meta-regression; S1 Appendix p52). Therefore, the level of significance of the pooled effect size of each antiplatelet agent is expected to vary according to the risk of events in the control arms. For example, the posterior effect size of aspirin was found insignificant in the un-adjusted analysis (RR:0.92; 95%CrI: 0.80–1.06), but in the baseline risk-adjusted analysis (pairwise RR centered on the average observed 5% risk of events) aspirin was found to be borderline effective (RR:0.85; 95%CrI: 0.75–1.01). The hierarchy of treatments according to the respective estimated risk thresholds of efficacy is shown in Fig 9 for the composite and each individual secondary outcome measure.

The order of effectiveness was similar to the comparative efficacy results of the primary network meta-analysis. Clopidogrel monotherapy performed best in reducing the composite rate major cardiovascular events in PAD patients and reached the threshold of significance at a composite risk level of 1.3 events per 100 person-years. Ticlopidine performed best in preventing cardiovascular deaths at a risk level of 1.1 events per 100 person-years. ADP antagonists were best in preventing non-fatal MIs at a risk level as low as 0.5–0.8 events per 100 person-years. Finally, aspirin and Clopidogrel (as monotherapies or in combination) performed best in prevention of non-fatal strokes becoming significant at a risk level of 0.7–0.8 events per 100 person-years (Fig 9).

### Consistency and heterogeneity

Overall, the network of evidence was robust and loop-specific inconsistencies were identified only when examining the closed loops of Cilostazol and Picotamide in case of non-fatal stroke (S1 Appendix p 53). The complete results of the direct and mixed treatment comparisons are outlined in detail in the S1 Appendix (pp 44–50) for all outcome measures. Complete numerical results of the baseline risk adjusted model are provided along with the standard models for comparison purposes. Results from direct frequentist pairwise comparisons (fixed and random effects models) aligned well with those obtained from the network meta-analysis both in magnitude and direction with only minor differences (S1 Appendix pp 44–50). The high level of correlation between the fixed and random effects models of the frequentist approach was in agreement with the prior hypothesis of homogeneity for the Bayesian approach of mixed treatment comparisons. There was no publication bias after visual inspection of the respective funnel plots of all outcome measures (S1 Appendix pp 26–42).

Results were stable in the various sensitivity analyses apart from minor numerical differences (S1 Appendix pp 26–42). Exclusion of the 2 primary prevention trials with aspirin did not have a significant impact on either Aspirin itself or the rest of indirect comparisons. The majority of low quality trials were identified in the comparisons of aspirin and Ticlopidine versus placebo. Exclusion of low-quality trials did not have a significant impact in the pooled summary estimates. Aspirin was found to be effective only after limiting the evidence to high quality secondary prevention trials (RR: 0.46; 95%CI: 0.24–0.88, I^2^ = 0%), but the result may not be reliable as it was primarily driven by the inclusion of a single factorial trial; CLIPS [35] (RR: 0.35; 95%CI: 0.14–0.84).

## Discussion

Risk factor modification and antiplatelet treatment are recommended for patients with symptomatic PAD with the aim of reducing the risk of future MACE and improve outcomes following lower limb revascularization.[4, 8, 36] PAD patients typically suffer from advanced multi-organ atherosclerosis and >50% of those will exhibit concomitant coronary artery or cerebrovascular disease.[37] Patients who suffer from a combination of coronary artery disease (CAD) and intermittent claudication (IC) also appear to have higher levels of inflammatory and prothrombotic biomarkers than patients with CAD alone.[10, 38] This combined with evidence to suggest under-prescription of evidence-based medical therapies[37] and the association of lower extremity PAD with the poorest clinical outcomes, especially in the presence of diabetes,[39] suggests that PAD patients may be a high-risk subgroup, who are most susceptible to MACE. The Antithrombotic Trialists’ Collaboration highlighted a 23% reduction of cardiovascular events with use of aspirin in a PAD subset, but two thirds of the included trials actually evaluated antiplatelets other than aspirin.[9] Aspirin is, however, used as the mainstream antiplatelet agent in patients with cardiovascular disease and concomitant PAD symptoms, despite a paucity of evidence for using this specific medication.[10, 40]

In order to better inform medical decision making for treatment of the PAD population, we synthesized comparative evidence from 49 different RCTs and carried out a network meta-analysis within a Bayesian framework to compare the efficacy of different antiplatelet agents. To our knowledge, this is the first comprehensive network meta-analysis of different antiplatelet options tested for prevention of MACE, as well as leg amputations in patients suffering from PAD. Within this large investigation, we have shown that aspirin, Cilostazol, Vorapaxar and Picotamide were largely ineffective in protecting PAD patients from adverse cardiovascular events and/or amputations. Only ADP antagonists (Ticlopidine, Clopidogrel, Clopidogrel plus aspirin, and Ticagrelor plus aspirin) achieved a significant reduction of the composite endpoint compared with placebo. Upon scrutiny of the individual secondary outcome measures, Ticlopidine monotherapy is the only therapy that can significantly reduce cardiovascular deaths, but it has been gradually withdrawn from clinical use because of a higher incidence of haematological disorders.[11] ADP inhibitors are the best treatment to prevent myocardial infarction with a 28% event rate reduction, and aspirin had the strongest protective effect against stroke with a 27% rate reduction.

Only a small number of trials were designed to examine primary prevention in PAD patients. The majority of trials included patients with intermittent claudication and nearly all trials published within the last decade included cases with either open surgical or endovascular limb revascularization. As a result, the observed baseline cardiovascular risk varied widely among different trials. We therefore introduced a regression adjusted analysis according to trial-specific baseline risk as a surrogate of patient heterogeneity. Results of the adjusted analysis were largely similar to the unadjusted primary analysis, but the hierarchical risk stratification analysis highlighted certain scientific observations. Aspirin monotherapy is effective only beyond a very high cardiovascular risk threshold (above 5.1%). Clopidogrel monotherapy is effective even in cases with low- to intermediate-cardiovascular risk (above 1.3%).

In terms of safety, the combination of Vorapaxar and aspirin was related with an increased risk of bleeding (NNH = 130), as was Clopidogrel and aspirin (NNH = 215). Overall, Clopidogrel monotherapy was associated with the most favourable benefit-harm profile (79% cumulative rank probability best and 77% cumulative rank probability safest). We therefore propose that Clopidogrel should be used for secondary prevention of adverse events in PAD patients and for primary prevention in asymptomatic patients with a low ABI at high risk of developing PAD. As PAD patients often have a complex past medical history, the risk-adjusted hierarchies of different antiplatelets may also serve as a useful guide for choosing between different agents according to individual patient characteristics, projected level of cardiovascular risk and estimated risk of bleeding. [17, 39] Aspirin, for example, may have a role in protecting PAD patients who have a higher risk of cerebrovascular events due to carotid stenosis.

Another finding from this study is the confirmation that dual antiplatelet therapy with a combination of Clopidogrel and aspirin can reduce major leg amputations following revascularization (32% reduction of event rates compared to Aspirin monotherapy; NNT = 94). This finding was the result of the pooled analysis of 3 RCTs with 3,527 patients and more than 8,000 person-years of follow-up including both surgical and endovascular revascularizations. The use of dual antiplatelet therapy with Clopidogrel and aspirin will be associated with an increased risk of bleeding, but as the number of major leg amputations avoided will be greater than the number of severe bleeding events induced, we postulate that dual antiplatelet therapy should be judiciously prescribed following lower limb revascularization.

There are some limitations to the present analysis. First, by design network meta-analyses are prone to uncertainty and potential bias, which may compromise the accuracy of the network of evidence. Network meta-analyses often have an explanatory character and primarily aim to identify areas of scientific uncertainty or to inform preliminary analyses during the design phase of proper randomized controlled trials. Point estimates of certain nodes may be derived mostly by indirect evidence and should be confirmed by future trials. Sensitivity and consistency analyses have, however, shown the robustness of the results and the presented network of evidence was well connected around Aspirin as the main comparator. Second, a fixed effects model was chosen over random effects because of significant discrepancies between frequentist and Bayesian results. A Bayesian random effects model produced unwarranted heterogeneity between trials. As the results of the direct fixed and random effects models were nearly identical in the majority of comparisons (S1 Appendix pp 26–42), however, a posterior confirmation of homogeneity was demonstrated. Furthermore, meta-regression was employed to address baseline risk variability and produce risk stratified hierarchies of antiplatelet comparative efficacy. Finally, the network of evidence included RCTs published over the past 40 years. Improvements in general medical management over time could not be accounted for.

In conclusion, ADP receptor inhibitors are the only group of antiplatelets that significantly prevent major cardiovascular events in patients suffering from PAD. Clopidogrel monotherapy is associated with the most favourable benefit-harm profile, and dual antiplatelet therapy with a combination of aspirin and clopidogrel can reduce the rate of major amputations following revascularization, though carries an increased risk of severe bleeding. The present results could help with the design of large-scale clinical trials investigating ADP antagonists for primary prevention of adverse cardiovascular events.

### Author contributions

All authors have made significant contributions to the submitted work by participating in the conceptualization of the present meta-analysis, selection of the included trials and abstraction of the relevant data, drafting, revision and final approval of the submitted manuscript. The corresponding author was personally responsible for all Bayesian statistical modelling and preparation of the initial manuscript draft. All authors meet authorship criteria according to the ICMJE recommendations: (1) substantial contributions to conception and design, acquisition of data, or analysis and interpretation of data; 2) drafting the article or revising it critically for important intellectual content; 3) final approval of the version to be published; and 4) agreement to be accountable for all aspects of the work in ensuring that questions related to the accuracy or integrity of any part of the work are appropriately investigated and resolved.

## Supporting Information

S1 Appendix Supporting information. Supplemental material containing extended methods description (pp 2–12), detailed Trial Networks (pp 13–15), characteristics of all included randomised clinical trials (pp 16–25), all pairwise frequentist forest and funnel plots (pp 26–42), the estimated SUCRA hierarchies (p 43), detailed sensitivity analyses (pp 44–50), and results of model fit, meta-regression and consistency analysis (pp 51–53).
(DOCX)

S2 Appendix PRISMA Checklist. Preferred reporting items and scientific methods for the present systematic review and meta-analysis according to the PRISMA statement 2009.
(DOC)
