# Supplementary material for: Comparative Efficacy and Safety of Different Antiplatelet Agents for Prevention of Major Cardiovascular Events and Leg Amputations in Patients with Peripheral Arterial Disease: A Systematic Review and Network Meta-Analysis
Source: PLoS One. 2015 Aug 14;10(8):e0135692. doi: 10.1371/journal.pone.0135692 (PMC4537264; doi:10.1371/journal.pone.0135692)
Supplement: S1 Appendix — Supplemental material containing extended methods description (pp 2–12), detailed Trial Networks (pp 13–15), characteristics of all included randomised clinical trials (pp 16–25), all pairwise frequentist forest and funnel plots (pp 26–42), the estimated SUCRA hierarchies (p 43), detailed sensitivity analyses (pp 44–50), and results of model fit, meta-regression and consistency analysis (pp 51–53). (DOCX) [file pone.0135692.s001.docx]

**S1 APPENDIX**

**Comparative efficacy and safety of different antiplatelet agents for prevention of major cardiovascular events and leg amputations in patients with peripheral arterial disease: a systematic review and network meta-analysis**

**APPENDIX 1. Supplemental methods (pp 2-12)**

**APPENDIX 2. PRISMA flowchart and Trial Networks (pp 13-15)**

**APPENDIX 3. Included randomised clinical trials (pp 16-25)**

**APPENDIX 4. Frequentist forest and funnel plots (pp 26-42)**

**APPENDIX 5. SUCRA hierarchies (p 43)**

**APPENDIX 6. Sensitivity analyses (pp 44-50)**

**APPENDIX 7. Model fit, regression and consistency analysis (pp 51-53)**

**APPENDIX 1. Full methods**

*Search and selection*

This systematic review has been registered and published in the PROSPERO public database (CRD42014010299; http://www.crd.york.ac.uk/PROSPERO). We first identified and collated a list of randomized trials evaluating different antiplatelet medications in the PAD population from previous relevant meta-analyses.[[1-7](#_ENREF_1)] Further electronic searches of PubMed (Medline, 1950 to present), EMBASE (Excerpta Medical Database, 1980 to present), AMED (Allied and Complementary Database, 1985 to present), Scopus (1970 to present), the CENTRAL (Cochrane Central Register of Controlled Trials), archives of regulatory authorities (FDA, EMA, MHRA), the DARE and PROSPERO databases of meta-analyses, online repositories and proceedings of international conferences were then performed.

There were no restrictions on language, date or type of publication. Published and unpublished randomised trials with an open-label, single-blinded or double-blinded designs were assessed. Reference lists of included studies, previous meta-analyses and relevant narrative reviews were also interrogated. For the purpose of the present systematic review, the search focused on RCTs comparing any kind of antiplatelet agent (aspirin, ADP receptor antagonists, phosphodiesterase inhibitors, thromboxane inhibitors, protease activated receptor antagonists or other) versus placebo or with each other, for prevention of major cardiovascular events and/or leg amputations in the PAD population. The trial selection process complied with the Preferred Reporting Items for Systematic reviews and Meta-Analyses (PRISMA) statement. [[8](#_ENREF_8)]

Each study was evaluated for inclusion into the network meta­analysis using a structured checklist. Inclusion criteria were: (1) prospectively designed RCTs in which, antiplatelet treatments were randomised against placebo or another antiplatelet agent for primary or secondary prevention of cardiovascular events and/or amputations; (2) the target population included patients with PAD; (3) symptomatic and asymptomatic cohorts were allowed (4) follow-up of at least 12 weeks was available, and; (5) one or more of the selected primary and secondary endpoints were reported (see outcome measures below).

The literature search for eligible RCTs was last updated in May 2014 using combinations (Boolean syntax) of relevant medical terms and keywords along with corresponding Medical Subjects Headings (MeSH) as follows: Meta-analysis, Cochrane, Randomized, Controlled trial, Peripheral arterial disease, Bypass surgery, Percutaneous Angioplasty, Aspirin, Clopidogrel, Ticlopidine, Ticagrelor, Dual antiplatelet therapy, Amputation, Cilostazol, Picotamide, Vorapaxar, Stroke, Myocardial infarction, Death, Composite, Prevention, Diabetes and Claudication.

*Endpoints and abstraction*

Data were extracted from the text, survival curves and tables of published manuscripts or from meeting abstracts and presentations indexed online following international conferences and symposia. The characteristics of the enrolled populations, definitions of endpoints, and number of accrued events during follow-up were entered on to a bespoke abstraction form. Trial investigators were contacted regarding any missing or conflicting data and outcome measures missing from primary publications were extracted from other previous meta-analyses and cross-referenced as necessary.[[2](#_ENREF_2),[3](#_ENREF_3),[6](#_ENREF_6),[7](#_ENREF_7)] Evaluation of the quality of the RCTs included in the network meta-analysis was performed using the Jadad 5-point instrument for assessing risk of bias in randomised controlled trials.[[9](#_ENREF_9)] The same tool has been used in previous systematic reviews of aspirin.[[6](#_ENREF_6)] Any disagreements were resolved by consensus.

An intention-to-treat principle was followed for analysis of primary and secondary endpoints. Primary efficacy endpoints were the rate of major cardiovascular events and, the rate of major leg amputations. Major cardiovascular events included all deaths from vascular causes, numbers of non-fatal myocardial infarction (MI) and numbers of non-fatal stroke. Secondary efficacy endpoints included all of the individual components from the primary composite endpoint. Major leg amputations included any amputation above the ankle and were analysed separately as they were not uniformly reported by the majority of the trials. The primary safety endpoint was the rate of severe or major bleeding as reported by each study.

The network of evidence was designed by assigning separate nodes to each individual treatment. Monotherapies were treated separately from combination therapies. The only exception was dipyridamole, which was pooled together with aspirin to maintain consistency with the design and results of older meta-analyses.[[2](#_ENREF_2),[6](#_ENREF_6)] Different aspirin doses were grouped together as outcomes have been shown to be similar. In multi-arm studies investigating different doses of the same drug versus placebo or with factorial study designs, the arms of active antiplatelet therapy were pooled together with the assumption that were no drug-drug interactions. Apart from the extended network of individual therapies, a condensed secondary network to investigate the comparative efficacy and safety of different drug classes (with or without aspirin) was constructed. Trials that compared different agents from the same class (ADP inhibitors) were excluded from the class analysis. The corresponding author had access to the whole dataset, performed all statistical analyses and has final overall responsibility for the submitted version of the manuscript. There was no funding source for this study.

*Statistical methods*

First, direct pairwise meta-analyses of head-to-head comparisons were performed with standard frequentist inference methods (Fixed Mantel-Haenszel [M-H] or random DerSimonian and Laird [D-L] effects, RevMan software [Review Manager Version 5.1; The Cochrane Collaboration, Copenhagen, Denmark] and the Statsdirect statistical package [Version 2.7.9, Statsdirect Ltd, Cheshire, United Kingdom]) as necessary. Second, quantitative data synthesis of both the extended and condensed networks of RCTs was performed with Bayesian inference using Gibbs sampling methods that allow calculation of all possible direct and indirect mixed treatment comparisons (WinBUGS 1.4.3, MRC Biostatistics Unit, Cambridge, United Kingdom). In case of zero cells in 2x2 contingency tables, continuity correction was applied with the addition of 0.5 to each observed frequency in order to avoid statistical artefacts. Design and Bayesian hierarchical modeling of the present network meta-analysis complied with the guidelines of the National Institute for Health and Excellence Decision Support Units (NICEDSU). [[10-13](#_ENREF_10)]

Summary statistics of relative treatment effects are reported as the median and accompanying 95% Credibility Intervals (95% CrI) of the posterior distribution. CrIs serve the same purpose as confidence intervals in frequentist statistics. In case of Bayesian inference 95% CrI that do not cross unity (for relative effects) or zero (for absolute effects) are considered significant and would be equivalent to a significance of α=0.05 in case of frequentist inference. Absolute treatment effects were calculated on the natural scale following an exploratory analysis of the placebo control arms. Cardiovascular deaths, non-fatal MIs, non-fatal strokes, major amputations, and severe bleeding events were recorded as counts of events per 100 person-years (Event Rates) to account for the variable follow-up period between RCTs and analysed with an MTC fixed effects Poisson model to calculate pairwise Rate Ratios (RR) between different treatments. Posterior medians (95% CrI) of how each treatment compares separately to placebo (reference) and aspirin are reported.

The probability of each treatment being the best (lowest rate of MACE) and/or safest (lowest rate of bleeding events), the number-needed-to-treat (NNT; 95% CrIs), and the number-needed-to-harm (NNH; 95% CrIs) were also calculated to provide measures of treatment efficacy. We constructed rankograms of cumulative rank probabilities of how each treatment ranks against each other in terms of being the 1^st^, 2^nd^, 3^rd^, etc best treatment option. In addition, we present a hierarchy of the efficacy and safety of the various antiplatelet treatments based on their cumulative rank probabilities and the Surface Area Under the Cumulative Rankograms (SUCRA, %) as proposed by Salanti et al. [[14](#_ENREF_14)]

*WinBUGS modelling*

WinBUGS code was written and adapted according to recommendations of the NICE Decision Support Units (<http://www.nicedsu.org.uk>).[[13](#_ENREF_13),[15](#_ENREF_15)] Bayesian inference with WinBUGS employs Markov Chain Monte Carlo (MCMC) simulation to calculate the posterior distributions of the interrogated nodes within the framework of the chosen model and likelihood function on the basis of prior assumptions. For the purposes of this analysis we first fitted a Bayesian hierarchical model for multiple comparisons of different treatment options using placebo as the treatment of reference.[[15](#_ENREF_15)] Posterior medians (95% CrI) of the point estimates against placebo and aspirin were calculated (models available on request by the authors).

Following exploratory direct comparisons with random and fixed effects models, we found that there was universally very good agreement between the point estimates of the two models and minimal heterogeneity was found in nearly all subgroup analyses in the whole dataset. A fixed effects inverse variance model was chosen to account for the small number of studies informing most of the pairwise comparisons of the present MTC. A fixed effects inverse variance model has been also proposed as a better approach in case of synthesis of trials with low rates of events and zero total events trials.[[16](#_ENREF_16)] The latter were included to reduce uncertainty around the pooled estimates and decrease between-study heterogeneity, and can be readily modelled within a Bayesian framework without the need for continuity corrections.[[16](#_ENREF_16)]

In case of multiple treatments analysis of the present network, a Bayesian random effects model either failed to reach convergence because of overparameterisation or underpowered the analysis so that it did not identify significant differences in case of treatment contrasts that were significantly different in both the fixed and random effects frequentist models. A poor model fit occurred even when using informative priors for between study heterogeneity. Therefore, for the mixed treatments comparison the random effects model was abandoned in favour of a fixed effects model under the assumption of homogeneity between different studies and considering the small number of studies informing most of the comparisons.

Because of conceptual differences in study designs and baseline demographics of recruited cohorts, the observed baseline risk of cardiovascular events varied widely between the reference treatment arms. Treatment effects may vary according to patient-level variables or trial-specific characteristics. Baseline risk is a proxy for unmeasured but important patient-level characteristics that may relate to significant clinical heterogeneity. Naive approaches that adjust the observed risk of events in the control groups or the average risk of events in the control and treatment groups are flawed and may produce seriously misleading results. However, a Bayesian approach with Gibbs sampling (WinBUGS) has been recommended as a more appropriate method for investigating the relationship between treatment effects and underlying baseline risk across trials in meta-analyses and adjusting for baseline confounders.[[17](#_ENREF_17),[18](#_ENREF_18)] For a detailed description of Bayesian modelling methods to account for baseline risk in network meta-analysis, where it is of interest to adjust for baseline imbalances in order to minimize both heterogeneity and inconsistency, the reader may refer to other dedicated technical resources.[[12](#_ENREF_12),[17](#_ENREF_17),[18](#_ENREF_18)]

In the present study, the uncertainty and clinical heterogeneity introduced by the variable baseline characteristics of PAD cohorts was accounted for by extending the analysis to a meta-regression model on trial-specific baseline risk of the control arms.[[18](#_ENREF_18),[19](#_ENREF_19)] To better inform decision making and aid interpretation of the results from a clinical viewpoint, we did a hierarchical risk stratification analysis of the pooled treatment effects. We combined the meta-regression coefficients of baseline risk analysis with the uncertainty surrounding the posterior medians of the rate ratios of events for each treatment to calculate the level of risk where each treatment is projected to reach statistical significance (97.5% CrI of the posterior median crosses unity).

Reported posterior medians (95% CrI) are based on a standard Bayesian fixed effects models with Poisson likelihood, but further extensive meta-regression against baseline risk was performed to explore the relationship of the posterior distributions of the treatment effects with the observed baseline risk of events. Minimally informative priors for effect sizes and precisions were used for all calculations of the present analysis to avoid bias. Three Markov chains were compiled and run, while convergence was confirmed with the Brooks–Gelman–Rubin diagnostic tool [[20](#_ENREF_20)] and by inspection of history plots of monitored nodes. An initial burn-in simulation of 50,000 iterations was discarded and inference of final summary statistics was based on simulation of an additional 100,000 iterations.

*Heterogeneity, consistency and sensitivity analyses*

The validity and robustness of NMA depends largely on the distribution of effect modifiers (covariates) not only between studies with the same contrast (i.e. heterogeneity in case of standard pairwise meta-analysis), but also between different contrasts (i.e. inconsistency between direct and indirect contrast estimates).[[21](#_ENREF_21)] Any disagreement between the direct evidence available for a specific contrast and the indirect evidence inferred by the rest of the network would give rise to inconsistency.[[22](#_ENREF_22)] The issues of evaluating and minimizing uncertainty around calculated point estimates of NMAs inherently require probing for underlying heterogeneity and inconsistency of the whole network of evidence. The straightforward *Cochran’s Q (chi^2^)* and the *I^2^* statistical test were calculated to test for statistical evidence of heterogeneity between RCTs with the same control and active arms. Briefly, *I^2^* values <25% indicate low, 25% to 50% moderate, and >50% high heterogeneity [[23](#_ENREF_23)]. Small study effects and publication bias were evaluated by visual inspection of respective funnel plots. Funnel plots are plots of the trials' estimated effect sizes (RR) against the standard error of the log-transformed estimates (SE(log[RR])) and in the presence of publication or other bias they may appear to be skewed and asymmetrical [[24](#_ENREF_24)].

Consistency, sensitivity and metaregression analyses were performed to test the validity and robustness of the results. In case of MTC network meta-analyses, the risk of network inconsistency is greatly reduced if between trial heterogeneity is low. [[25](#_ENREF_25)] To exclude any node-specific discrepancies, pairwise direct and indirect effect estimates of closed loops of evidence were inspected for any disagreement. Loop-specific inconsistency factors were derived with the Bucher’s back-calculation method. [[25](#_ENREF_25),[26](#_ENREF_26)] In cases of frequentist modelling, sensitivity analysis examined the random (DerSirmonian and Laird) versus fixed effects (inverse variance) model for all direct pairwise comparisons (Appendix). Application of a random effects model was employed to potentially account for inter- trial heterogeneity in calculating the pooled effect estimates. Frequentist sensitivity analysis was used to check for significant deviation in the direction and magnitude of the results in case of exclusion of the 2 large Aspirin primary prevention trials,[[27](#_ENREF_27),[28](#_ENREF_28)] and in case of exclusion of low to medium quality RCTs (Jadad score 1-3). Leave-one-out meta-analysis was also performed in case of direct syntheses involving more than 3 individual trials to check for high dependency on an individual dataset.

Using Bayesian modelling, standard and baseline-risk adjusted models were compared. Sensitivity analysis included the calculation of the posterior medians having centered on the mean value of covariate baseline risk as described. [[18](#_ENREF_18),[19](#_ENREF_19)] Global model fit and parsimony was compared between different fitted models (standard fixed effects and baseline-risk adjusted fixed effects) in order to decide on the most accurate model. Goodness of fit was compared with the posterior mean of the total residual deviance and the Deviance Information Criterion (DIC) criterion. [[15](#_ENREF_15)] For a good model fit the residual deviance must be close to the total number of study arms analysed and the model with the lowest DIC is preferred. The level of statistical significance was set at α=0.05 for frequentist inference, while results associated with 95% CrI that do not cross unity (for relative effects) or zero (for absolute effects) were considered significant in case of Bayesian inference. Two-tailed posterior probabilities under minimally informative priors were calculated in WinBUGS to approximate frequentist P values and aid interpretation of the posterior medians of the point estimates.[[29](#_ENREF_29)]

**APPENDIX 2. PRISMA flowchart and Trial Networks**


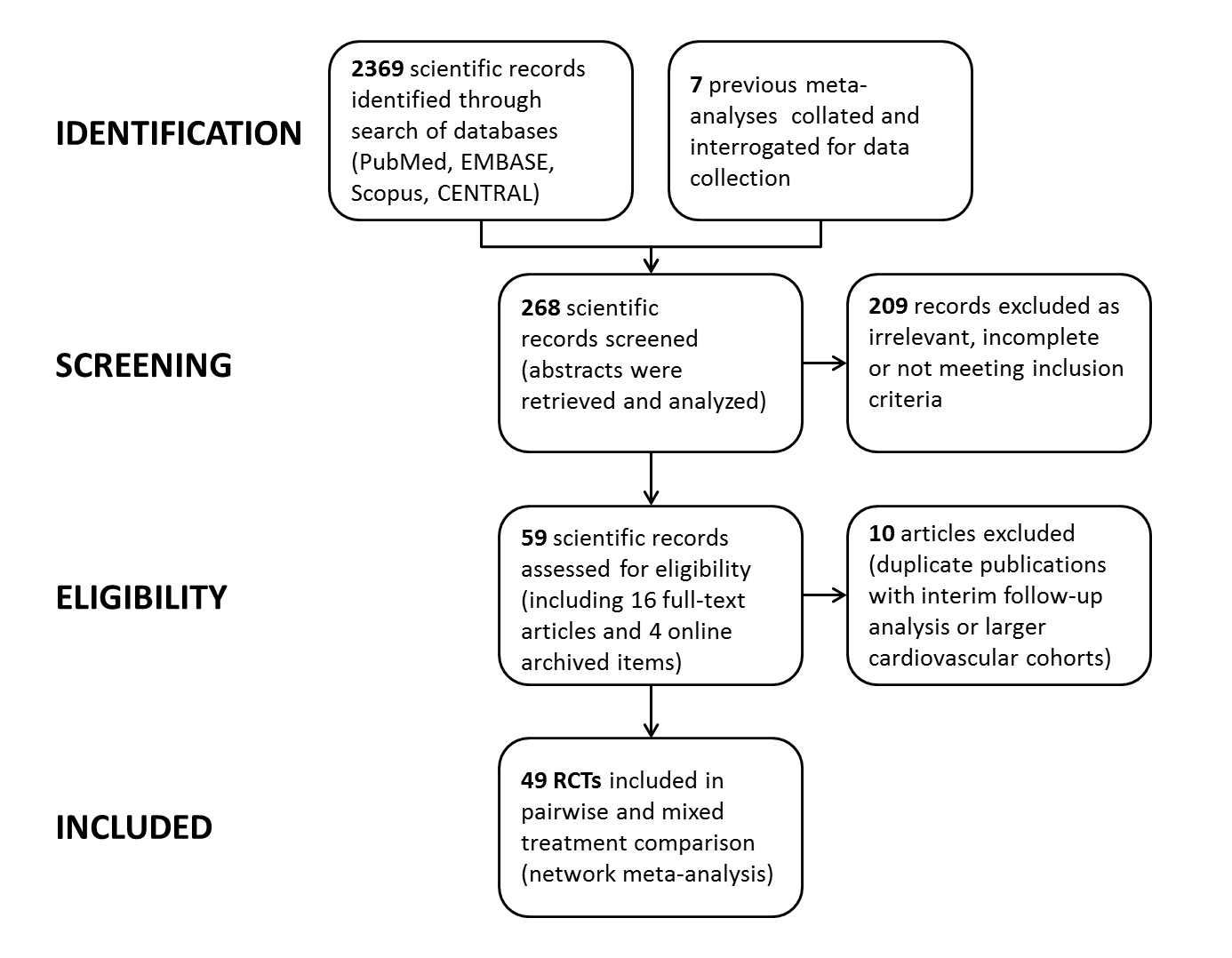


**Trial selection process according to the PRISMA statement.**


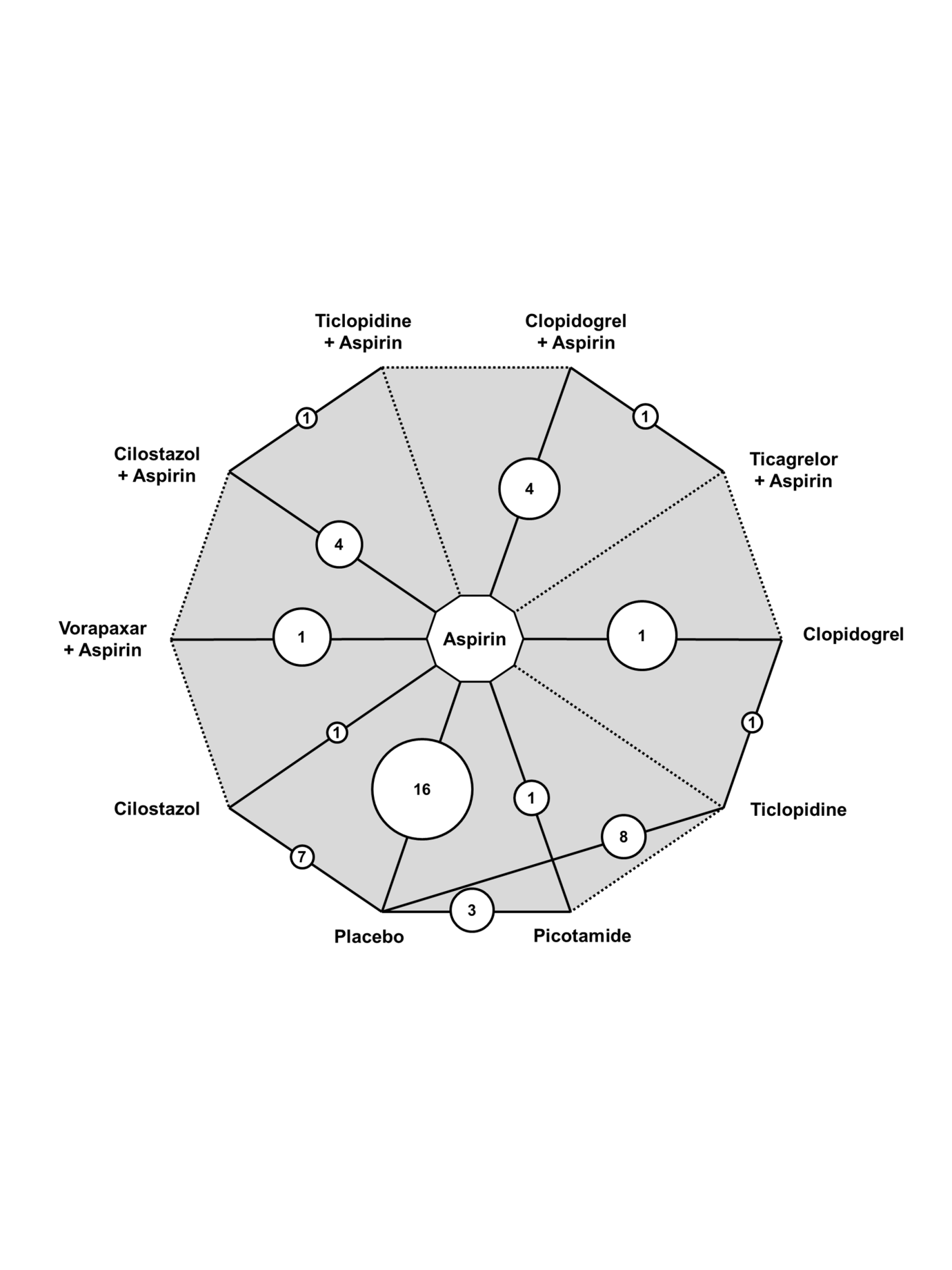


**Full primary network of evidence.** Straight lines denote direct head-to-head comparisons and dotted lines denote indirect comparisons where direct comparison data is missing. Numbers refer to the number of RCTs with direct comparisons available for each link and the size of circles is proportional to the pooled sample size (person-years) available for each direct comparison.


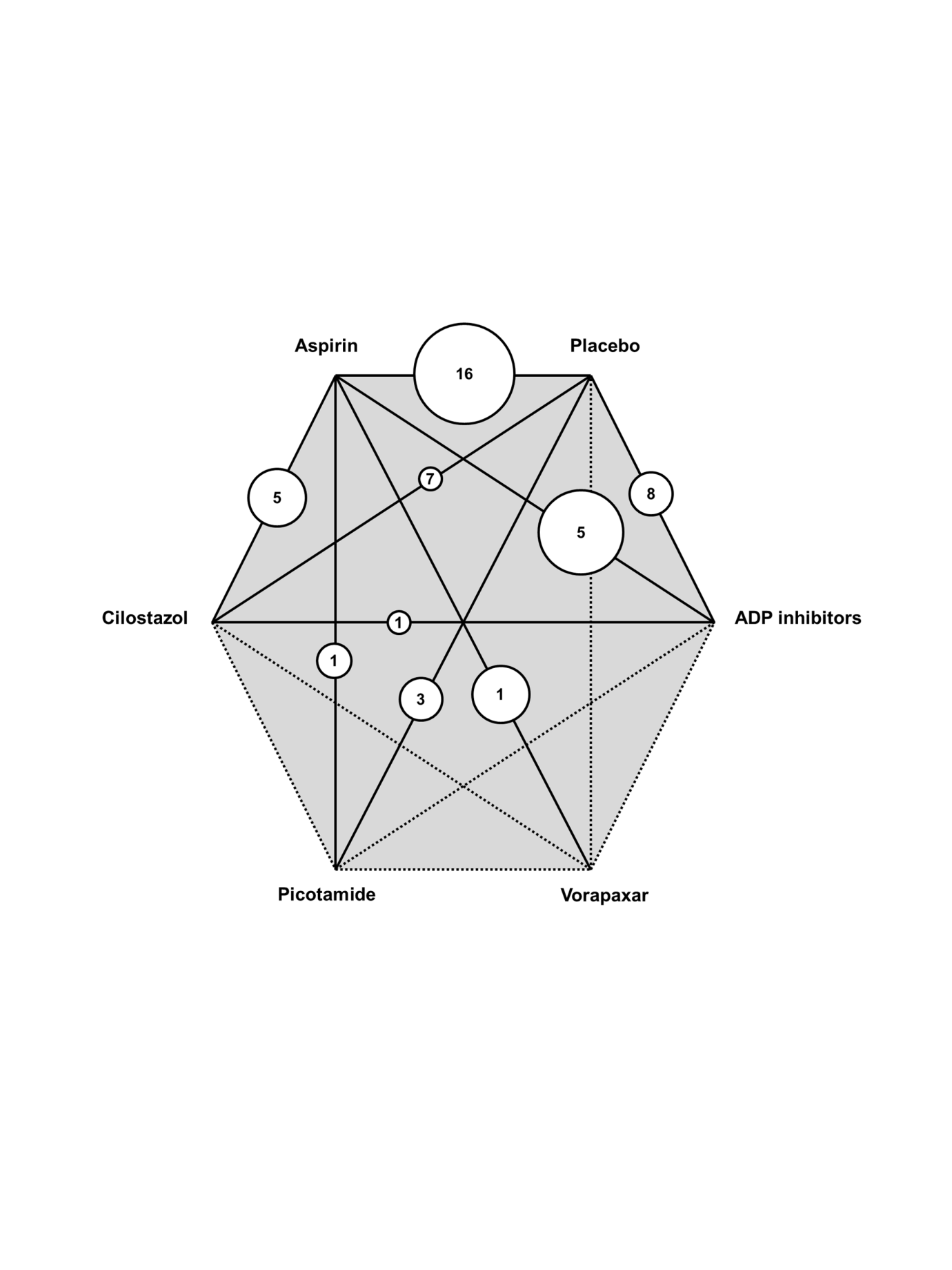
 **Condensed secondary network of evidence (class level).** Straight lines denote direct head-to-head comparisons and dotted lines denote indirect comparisons where direct comparison data is missing. Numbers refer to the number of RCTs with direct comparisons available for each link and the size of circles is proportional to the pooled sample size (person-years) available for each direct comparison.

**APPENDIX 3. Included randomised clinical trials**

| **Table 1. Characteristics of included randomized controlled trials** | | | | | | |
| --- | --- | --- | --- | --- | --- | --- |
| **Reference and study year** | **Total number of patients recruited** | **Target patient population** | **Active drug interventions** | **Study duration or median follow-up** | **Trial quality (Jadad score[**[**9**](#_ENREF_9)**])** | **Extra notes on study design and analysis** |
| ***Aspirin versus Placebo*** | | | | | | |
| Munich-A,[[30](#_ENREF_30)] 1975 | n=176 | Patients with intermittent claudication | Aspirin 1,500mg versus placebo | 2 years | - | Concealment of randomization unclear |
| Munich-B,[[30](#_ENREF_30)] 1975 | n=82 | Patients with intermittent claudication | Aspirin 1,500mg versus placebo | 2 years | - | Concealment of randomization unclear |
| Loew et al.[[31](#_ENREF_31)]  1977 | n=428 | Infrainguinal bypass grafts for occlusive disease | Aspirin 1,500mg versus placebo | 1 year | - | Patency of peripheral bypass grafts studied |
| Green et al,[[32](#_ENREF_32)]  1982 | n=49 | Patients with infrainguinal synthetic ePTFE bypass grafts | Aspirin 325mg versus Aspirin 325mg and dipyridamole 75mg  versus placebo  3 times daily | 1 year | 3 | Single centre  3-arm study; Aspirin arms pooled together for analysis against placebo |
| Schoop-I,[[33-35](#_ENREF_33)]  1984 | n=300 | Men with symptomatic intermittent claudication because of unilateral femoral stenosis | Aspirin 330mg versus Aspirin 330mg and dipyridamole 75mg  versus placebo  3 times daily | 5 years | - | Concealment of randomization unclear.  3-arm trial; Aspirin arms pooled together for analysis against placebo |
| Kohler et al.[[36](#_ENREF_36)]  1984 | n=100 | Patients with infrainguinal PTFE or autologous veinbypass grafts | Aspirin 325mg and dipyridamole 75mg  versus placebo  3 times daily | 2 years | 3 | Patency of peripheral bypass grafts studied |
| Goldman and McCollum,[[37](#_ENREF_37)]  1984 | n=53 | Patients with Dacron/PTFE femoropopliteal bypass grafts | Aspirin 300mg and dipyridamole 75mg  versus placebo  3 times daily | 1 year | 4 | Patency of peripheral bypass grafts studied |
| Donaldson et al.[[38](#_ENREF_38)]  1985 | n=65 | Femoropopliteal synthetic Dacron bypass grafts for disabling claudication | 330mg and dipyridamole 75mg  versus placebo  3 times daily | 1 year | 3 | Concealment of randomization unclear |
| Hess et al,[[39](#_ENREF_39)] 1985 | n=240 | Stable intermittent claudication and angiographically proven peripheral arterial disease of the lower extremities | Aspirin 330mg versus Aspirin 330mg and dipyridamole 75mg  versus placebo  3 times daily | 2 years | 4 | Serial arteriograms taken.  3-arm trial; Aspirin arms pooled together for analysis against placebo |
| Veterans Administration Cooperative Study,[[40](#_ENREF_40)]  1986 | n=231 | Diabetic men with either a recent major amputation for gangrene or active gangrene | Aspirin 325mg and dipyridamole 75mg  versus placebo  3 times daily | 42 months | 3 | Outcomes on contralateral leg amputations available |
| Heiss et al.[[41](#_ENREF_41)]  1990 | n=199 | Patients who underwent percutaneous angioplasty for >50% stenosis of the femoropopliteal artery | Aspirin 330mg and dipyridamole 75mg  versus Aspirin 100mg and dipyridamole 75mg  versus placebo  3 times daily | 6 months | 4 | 45 out of the 199 did not complete the 6-month trial period.  3-arm trial; Aspirin arms pooled together against placebo |
| McCollum et al,[[42](#_ENREF_42)]  1991 | n=549 | Peripheral femoropopliteal vein bypass for claudication, rest pain or gangrene | Aspirin 300 mg and dipyridamole 150mg  versus placebo twice daily | 34 months | 3 | Life table analysis of graft failures.  Amputations not reported |
| BMFT-II,[[2](#_ENREF_2)]  1998 | n=334 | Patients who underwent percutaneous angioplasty of the peripheral arteries | Aspirin 325mg and dipyridamole 75mg  versus placebo  3 times daily | 3 years | - | Unpublished. Event counts extracted from previous collaborative meta-analysis[[2](#_ENREF_2)] |
| CLIPS,[[43](#_ENREF_43)] 2007 | n=366 | Symptomatic (claudicants) or asymptomatic patients with documented PAD (imaging and ABI<0.85 or toe index<0.6) | Aspirin 100mg once daily with or without antioxidants versus placebo ± antioxidants | 21 months | 4 | 2x2 factorial design – Aspirin arms pooled together for analysis against placebo/vitamin control arms  18% of target population enrolled |
| POPAPAD,[[27](#_ENREF_27)] 2008 | n=1,276 | Asymptomatic diabetics >40 years old with an ABI<0.99 (symptomatic cardiovascular disease excluded) | Aspirin 100mg once daily with or without antioxidants versus placebo ± antioxidants | 6.7 years | 5 | 2x2 factorial design – Aspirin arms pooled together for analysis against placebo arms |
| Fowkes et al,[[28](#_ENREF_28)] 2010 | n=3,350 | Asymptomatic men and women with a low ABI (≤0.95) and without any cardiovascular disease | Aspirin 100mg once daily versus placebo | 8.2 years | 5 | ABI screening of the general population in Scotland for primary prevention |
| ***Ticlopidine versus Placebo*** | | | | | | |
| Aukland et al,[[44](#_ENREF_44)]  1982 | n=65 | Men with atherosclerotic intermittent claudication and haemorheological abnormalities | Ticlopidine 250mg twice daily versus placebo | 1 year | 4 | Investigation of several prothrombotic plasma tests |
| Stiegler et al,[[45](#_ENREF_45)]  1984 | n=114 | Patients with intermittent claudication - Arterial angiography at baseline and at 1 year | Ticlopidine 250mg twice daily versus placebo | 13 months | 3 | Event counts extracted from previous collaborative meta-analysis[[2](#_ENREF_2)] |
| Castelli et al.[[46](#_ENREF_46)]  1986 | n=50 | Femoropopliteal thromboendarterectomy for arterial occlusive disease | Ticlopidine 250mg twice daily versus placebo | 6 months | 3 | Primary endpoint set on patency |
| US-ticlopidine,[[47](#_ENREF_47)]  1986 | n=203 | Patients with chronic intermittent claudication | Ticlopidine 250mg twice daily versus placebo | 6 months | - | Unpublished. Event counts extracted from previous collaborative meta-analysis[[2](#_ENREF_2)] |
| ACT,[[48](#_ENREF_48)]  1988 | n=169 | Chronic intermittent claudication (>1 year) due to peripheral vascular disease proven by angiography | Ticlopidine 250mg twice daily versus placebo | 6 months | 5 | Study prematurely terminated because of slow enrollment rate |
| Balsano et al.[[49](#_ENREF_49)]  1989 | n=151 | Patients with intermittent claudication (>6 months) and reduced ankle-brachial index | Ticlopidine 250mg twice daily versus placebo | 21 months | 4 | 3-month single-blind run-in period |
| STIMS,[[50](#_ENREF_50),[51](#_ENREF_51)]  1990 | n=687 | Patients with intermittent claudication and reduced ankle-brachial index | Ticlopidine 250mg twice daily versus placebo | 5.6 years | 5 | Swedish Ticlopidine Multicentre Study; Surgical reconstruction events published separately[[50](#_ENREF_50)] |
| EMATAP,[[52](#_ENREF_52),[53](#_ENREF_53)]  1994 | n=615 | Chronic intermittent claudication (>1 year) due to peripheral occlusive disease proven by imaging | Ticlopidine 250mg twice daily versus placebo | 6 months | 5 | Stratified multicenter RCT in Argentina  Diabetic and non-diabetic strata |
| ***Clopidogrel versus Aspirin*** | | | | | | |
| CAPRIE,[[54](#_ENREF_54)]  1996 | n=6,452 | Intermittent claudication and ABI≤0.85 or history of leg amputation, bypass surgery or angioplasty | Clopidogrel 75mg versus Aspirin 325mg once daily | 1.91 years | 5 | PAD subgroup out of 19,185 patients in total. 63% had undergone arterial intervention |

| ***Clopidogrel and Aspirin versus Aspirin*** | | | | | | |
| --- | --- | --- | --- | --- | --- | --- |
| CREDO,[[55](#_ENREF_55),[56](#_ENREF_56)]  2006 | n=272 | Patients referred for elective coronary angiography/angioplasty and documented extracardiac vascular disease  (in the primary study 86% eventually underwent a percutaneous coronary intervention) | Clopidogrel 75mg and Aspirin 325mg in both arms up to 28 days; clopidogrel 75mg and Aspirin 81-325mg versus placebo plus Aspirin 81-325mg (day 29 to 1 year) | 1 year | 5 | Subgroup analysis out of 2,116 patients; n=211 with documented PAD  (Loading dose of clopidogrel 300mg versus placebo peri-procedure) |
| CHARISMA,[[57](#_ENREF_57),[58](#_ENREF_58)]  2009 | n=3,096 | Asymptomatic PAD or intermittent claudication and ABI≤0.85 or history of leg amputation, bypass surgery or angioplasty | Clopidogrel 75mg plus low-dose aspirin 75–162 mg  versus placebo plus low-dose aspirin once daily | 28 months | 5 | Post-hoc analysis of the PAD subgroup out of 9,478 patients in total.  8.4% cases of the PAD subgroup were asymptomatic |
| CASPAR,[[59](#_ENREF_59)]  2010 | n=851 | Patients undergoing unilateral synthetic or autologous vein  below-knee bypass surgery for claudication, rest pain or gangrene | Clopidogrel 75mg plus aspirin  75–100mg  versus placebo plus aspirin  75–100mg  once daily | 1 year | 5 | 26-30% of the patients received heparin instead of Aspirin.  21-25% discontinued treatment. |
| MIRROR,[[60](#_ENREF_60),[61](#_ENREF_61)]  2012 | n=80 | Following percutaneous angioplasty with or without stenting of the femoropopliteal artery for claudication, rest pain or minor tissue loss | Aspirin  100 mg and clopidogrel 75 mg versus Aspirin 75mg and placebo once daily | 6 months | 5 | Single-centre; loading dose of Aspirin 500 mg and Clopidogrel 300 mg  Prior to intervention. Evaluated clopidogrel resistance |

| ***Ticagrelor and Aspirin versus Clopidogrel and Aspirin*** | | | | | | |
| --- | --- | --- | --- | --- | --- | --- |
| PLATO,[[62-65](#_ENREF_62)]  2011 | n=1,144 | Patients who presented with moderate- to high-risk acute coronary  Syndromes (ACS) and underwent coronary intervention or medical management  All patients also received Aspirin  75–100mg daily. | Ticagrelor 180mg  loading dose followed by 90mg twice daily versus  clopidogrel 300-600mg loading dose followed  by 75mg once daily for 6–12 months | 1 year | 5 | Post-hoc analysis of the PAD subgroup out of 18,624 patients in total. A quarter was diabetics and 6.1-6.2% of the whole cohort reported PAD symptoms  (prior revascularization or ABI < 0.9) |
| ***Clopidogrel versus Ticlopidine*** | | | | | | |
| COOPER,[[66](#_ENREF_66)]  2012 | n=431 | Patients with established PAD  (current intermittent claudication with an ABI <0.90 or  previous intervention in  a leg, such as angioplasty, atherectomy, bypass graft, or  other vascular intervention, including amputation) | Clopidogrel 75mg versus Ticlopidine 200mg once daily (for 12 weeks; then both arms assigned to Clopidogrel 75mg once daily up to 52 weeks) | 3 months | 5 | Japanese population with symptomatic PAD or following previous intervention.  12-week safety and primary outcome data analyzed only.  48% of patients received also cilostazol |
| ***Cilostazol versus Placebo (n=7)*** | | | | | | |
| 21-90-201 [[1](#_ENREF_1),[7](#_ENREF_7)]  Otsuka, 2002 | n=81 | Patients with PAD/IC (symptoms duration >6 months and ABI<0.90).  Rest pain and/or tissue loss excluded | Cilostazol 100mg twice daily versus placebo | 3 months | 5 | Combined meta-analytic report of n=8 studies funded by Otsuka Pharmaceuticals. |
| 21-92-202 [[1](#_ENREF_1),[7](#_ENREF_7),[67](#_ENREF_67)]  Otsuka, 2002 | n=516 | Patients with PAD/IC (symptoms duration >6 months and ABI<0.90).  Rest pain and/or tissue loss excluded | Cilostazol 50 or 100mg twice daily versus placebo | 6 months | 5 | Combined meta-analytic report of n=8 studies funded by Otsuka Pharmaceuticals 3-arm trial (Pooled analysis of the Cilostazol arms; no dose dependency) |
| 21-93-201 [[1](#_ENREF_1),[7](#_ENREF_7)]  Otsuka, 2002 | n=189 | Patients with PAD/IC (symptoms duration >6 months and ABI<0.90).  Rest pain and/or tissue loss excluded | Cilostazol 100mg twice daily versus placebo | 3 months | 5 | Combined meta-analytic report of n=8 studies funded by Otsuka Pharmaceuticals |
| 21-94-201 [[1](#_ENREF_1),[7](#_ENREF_7),[68](#_ENREF_68)]  Otsuka, 2002 | n=394 | Patients with PAD/IC (symptoms duration >6 months and ABI<0.90).  Rest pain and/or tissue loss excluded | Cliostazol 50 or 100mg twice daily versus placebo | 6 months | 5 | Combined meta-analytic report of n=8 studies funded by Otsuka Pharmaceuticals 3-arm trial (Pooled analysis of the Cilostazol arms; no dose dependency) |
| 21-94-203 [[1](#_ENREF_1),[7](#_ENREF_7)]  Otsuka, 2002 | n=239 | Patients with PAD/IC (symptoms duration >6 months and ABI<0.90).  Rest pain and/or tissue loss excluded | Cilostazol 100mg twice daily versus placebo | 4 months | 5 | Combined meta-analytic report of n=8 studies funded by Otsuka Pharmaceuticals |
| 21-94-301 [[1](#_ENREF_1),[7](#_ENREF_7)]  Otsuka, 2002 | n=247 | Patients with PAD/IC (symptoms duration >6 months and ABI<0.90).  Rest pain and/or tissue loss excluded | Cilostazol 100mg twice daily versus placebo | 6 months | 5 | Combined meta-analytic report of n=8 studies funded by Otsuka Pharmaceuticals |
| 21-95-201 [[1](#_ENREF_1),[7](#_ENREF_7)]  Otsuka, 2002 | n=215 | Patients with PAD/IC (symptoms duration >6 months and ABI<0.90).  Rest pain and/or tissue loss excluded | Cliostazol 100 or 150mg twice daily versus placebo | 3 months | 5 | Combined meta-analytic report of n=8 studies funded by Otsuka Pharmaceuticals 3-arm trial (Pooled analysis of the Cilostazol arms; no dose dependency) |

| ***Cilostazol versus Aspirin*** | | | | | | |
| --- | --- | --- | --- | --- | --- | --- |
| DAPC,[[69](#_ENREF_69)]  2010 | n=329 | Patients with type II diabetes who were suspected of peripheral arterial disease  (ABI<1.0 and/or poor pulses and/or clinical symptoms of PAD) | Cilostazol  100-200mg daily versus Aspirin  81-200mg daily | 2 years | 5 | Primary prevention of carotid atherosclerosis in East-Asian countries |
| ***Cilostazol and Aspirin versus Aspirin*** | | | | | | |
| CASTLE,[[70](#_ENREF_70)]  2008 | n=1,899 | Patients with a clinical diagnosis of PAD and symptomatic intermittent claudication | Cilostazol 100mg twice daily versus placebo. Majority of subjects in both groups also received Aspirin (72%). | 2 years | 5 | Around a quarter of the patients received also clopidogrel (27.4%). |
| Soga et al,[[71](#_ENREF_71)]  2009 | n=78 | Patients with IC due to femoropopliteal lesion and who underwent balloon angioplasty and provisional stent placement | Cilostazol 100mg twice daily and Aspirin (81-100mg) once daily versus Aspirin once daily | 2 years | 5 | Open label trial  All patients also on Ticlopidine 200mg/day prior to procedure, but stopped it up to 4 weeks post-procedure |
| STOP IC,[[72](#_ENREF_72)]  2013 | n=200 | Patients with symptomatic PAD (Rutherford stage >1) and who underwent balloon angioplasty and provisional stent placement of the femoropopliteal artery | Cilostazol 100mg twice daily and Aspirin 100mg once daily versus Aspirin100mg once daily | 1 year | 5 | Patients with stents were prescribed also on single thienopyridene for 1 month post-procedure to prevent stent thrombosis |
| 21-96-202 [[1](#_ENREF_1),[7](#_ENREF_7)]  Otsuka, 2002 | n=466 | Patients with PAD/IC (symptoms duration >6 months and ABI<0.90).  Rest pain and/or tissue loss excluded | Cilostazol 50 or 100mg twice daily versus Aspirin | 6 months | 5 | Combined meta-analytic report of n=8 studies funded by Otsuka Pharmaceuticals. In the control group 64% received Aspirin |

| ***Cilostazol and Aspirin versus Ticlopidine and Aspirin*** | | | | | | |
| --- | --- | --- | --- | --- | --- | --- |
| Iida et al,  2008 | n=127 | Patients who were successfully treated with balloon angioplasty and/or stent placement for femoropopliteal arterial occlusive disease | Cilostazol 200mg daily versus Ticlopidine 200mg daily (in addition to Aspirin100mg once daily in both groups) | 3 years | 4 | Open label, single institution |
| ***Vorapaxar and Aspirin versus Aspirin*** | | | | | | |
| TRA2°P-  TIMI 50,[[73](#_ENREF_73)] 2013 | n=3,787 | Patients with symptomatic and ABI<0.85 prior to leg revascularization (62% of the cases) | Vorapaxar 2.5mg daily versus matching placebo.  Majority of subjects in both groups also received Aspirin (88%). | 3 years | 5 | PAD subgroup analysis out of 26,449 subjects in total.  28% of the PAD cohort received dual antiplatelet therapy (Aspirin and thienopyridene) |
| ***Picotamide versus Placebo*** | | | | | | |
| Coto et al,[[74](#_ENREF_74)]  1989 | n=40 | Patients with peripheral occlusive arterial disease of the lower limbs at functional stage II of the Fontaine classification | Picotamide 300mg three times daily versus placebo | 6 months | 4 | Double-blind.  Concealment of randomization unclear |
| ADEP,[[75](#_ENREF_75)]  1993 | n=2,304 | Patients with intermittent claudication and ABI<0.85; with or without leg amputation and/or reconstructive vascular surgery | Picotamide 300mg three times daily versus placebo | 18 months | 5 | Double-blind randomized study in Italy. Patients on Aspirin or other antiplatelets were excluded |
| Neirotti et al,[[76](#_ENREF_76)]  1994 | n=20 | Patients with PAD functional stage 2 of the Fontaine classification and with intermittent claudication for at least six months | Picotamide 300mg three times daily versus placebo | 18 months | 4 | Double-blind.  Concealment of randomization unclear |

| ***Picotamide versus Aspirin*** | | | | | | |
| --- | --- | --- | --- | --- | --- | --- |
| DAVID,[[77](#_ENREF_77)]  2004 | n=1,209 | Patients with diabetes (>5 years) and diagnosis of PAD defined as at least 2 of the following: IC>2 months; loss of foot pulses; abnormal ABIs; previous leg amputation or bypass surgery; previous angioplasty | Picotamide 600mg twice daily versus Aspirin 320mg once daily | 2 years | 5 | Double-blind trial. Around 31% of the population was on insulin and 14% on statins |

**APPENDIX 4. Frequentist forest and funnel plots**

**COMPOSITE ENDPOINT – Fixed effects inverse variance model**

**COMPOSITE ENDPOINT – Random effects DerSimonian and Laird model**

**COMPOSITE ENDPOINT – funnel plot**

**CARDIOVASCULAR DEATHS – Fixed effects inverse variance model**

**CARDIOVASCULAR DEATHS – Random effects DerSimonian and Laird model**

**CARDIOVASCULAR DEATHS – funnel plot**

**NON-FATAL MI – Fixed effects inverse variance model**

**NON-FATAL MI – Random effects DerSimonian and Laird model**

**NON-FATAL MI – funnel plot**

**NON-FATAL STROKE – Fixed effects inverse variance model**

**NON-FATAL STROKE – Random effects DerSimonian and Laird model**

**NON-FATAL STROKE – funnel plot**

**MAJOR AMPUTATIONS – Fixed effects inverse variance model**

**MAJOR AMPUTATIONS – funnel plot**

**MAJOR BLEEDING – Fixed effects inverse variance model**

**MAJOR BLEEDING – Random effects DerSimonian and Laird model**

**MAJOR BLEEDING – funnel plot**

**APPENDIX 5. Efficacy hierarchies according to SUCRA (%).**


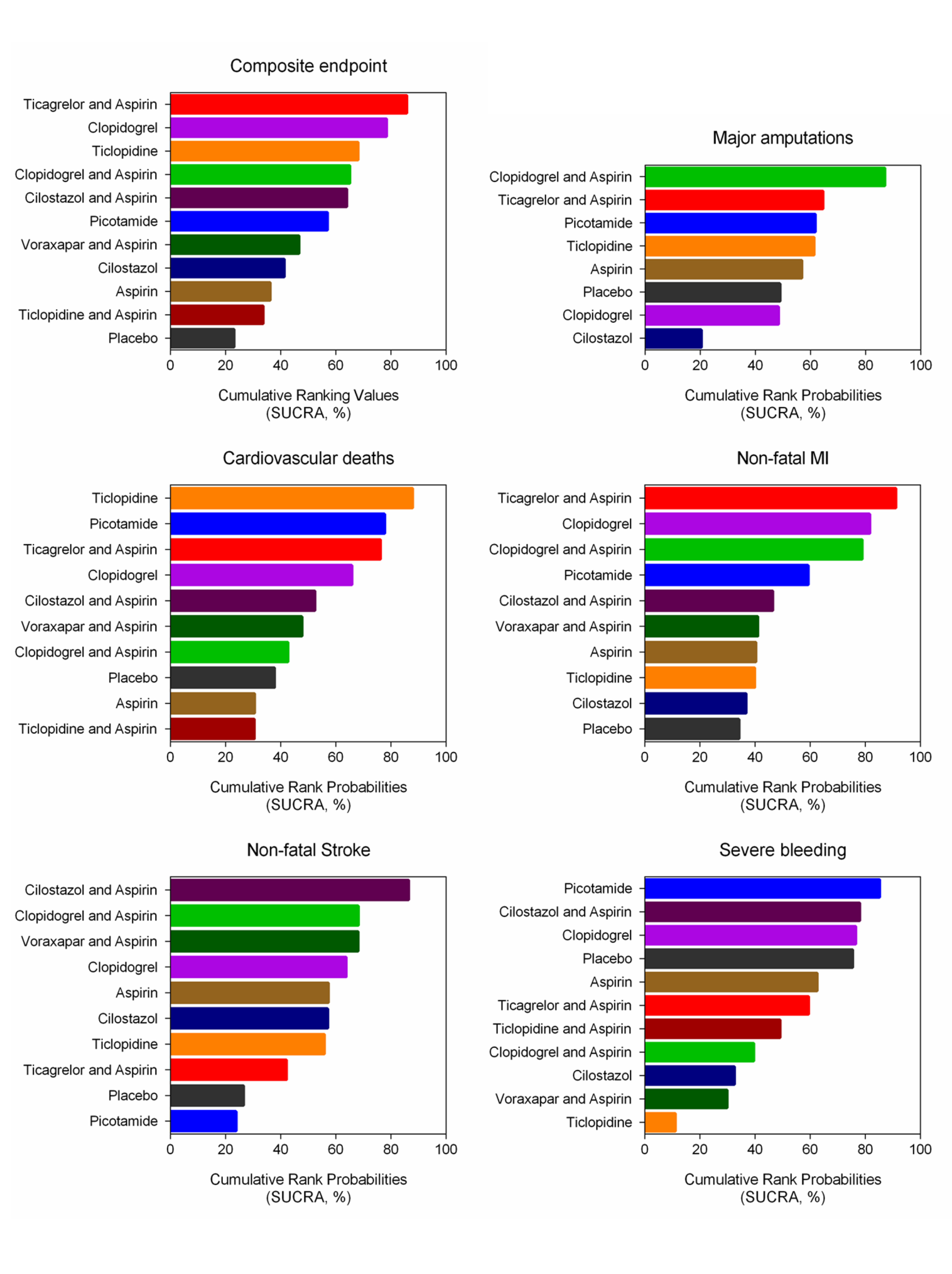


Different antiplatelet agents are ranked according to their SUCRA (%) values for each endpoint.

**APPENDIX 6. Sensitivity analyses**

Comparison of direct fixed and random effects frequentist models and Bayesian standard and risk-adjusted models

ASA denotes aspirin, TCD denotes Ticlopidine, CLP denotes Clopidogrel, TCG denotes Ticagrelor, CSZ denotes Cilostazol, VXP denotes Vorapaxar and PCD denotes picotamide

FE (IV) = Fixed effects inverse variance frequentist inference

RE (DL) = Random effects DerSimonian and Laird frequentist inference

FE (BY) = Bayesian fixed effects linear model

FE (RA) = Bayesian fixed effects linear model adjusted for trial-specific baseline risk

| **COMPOSITE**  **ENDPOINT** | **Placebo** | **ASA** | **TCD** | **CLP** | **CLP**  **+ASA** | **TCG**  **+ASA** | **CSZ** | **CSZ**  **+ASA** | **TCD**  **+ASA** | **VXP**  **+ASA** | **PCD** |
| --- | --- | --- | --- | --- | --- | --- | --- | --- | --- | --- | --- |
| **Placebo** | **FE(IV)**  **RE(DL)**  **FE(BY)**  **FE(RA)** | **0.92 (0.80-1.06) 0.92 (0.80-1.06)**  **0.92 (0.80-1.06)**  **0.85 (0.73-1.01)** | **0.77 (0.60-0.99)**  **0.77 (0.60-0.99)**  **0.75 (0.58-0.96)**  **0.77 (0.60-0.98)** | **-**  **-**  **0.72 (0.58-0.91)**  **0.67 (0.53-0.85)** | **-**  **-**  **0.78 (0.61-0.99)**  **0.72 (0.55-0.94)** | **-**  **-**  **0.67 (0.46-0.96)**  **0.62 (0.42-0.91)** | **0.85 (0.47-1.53)**  **0.85 (0.47-1.53)**  **0.94 (0.53-1.73)**  **0.97 (0.55-1.68)** | **-**  **-**  **0.77 (0.52 -1.15)**  **0.72 (0.48-1.08)** | **-**  **-**  **1.32 (0.29 -7.15)**  **1.24 (0.27-6.63)** | **-**  **-**  **0.87 (0.69-1.10)**  **0.81 (0.63-1.05)** | **0.83 (0.54-1.27)**  **0.83 (0.54-1.27)**  **0.82 (0.59-1.13)**  **0.75 (0.55-1.05)** |
| **ASA** |  | **FE(IV)**  **RE(DL)**  **FE(BY)**  **FE(RA)** | **-**  **-**  **0.81 (0.60-1.08)**  **0.90 (0.65-1.21)** | **0.77 (0.65-0.92)**  **0.77 (0.65-0.92)**  **0.78 (0.65-0.94)**  **0.78 (0.66-0.94)** | **0.84 (0.69-1.03)**  **0.84 (0.69-1.03)**  **0.84 (0.68-1.04)**  **0.84 (0.68-1.04)** | **-**  **-**  **0.72 (0.51-1.02)**  **0.72 (0.51-1.02)** | **2.10 (0.39-11.4)**  **2.10 (0.39-11.4)**  **1.02 (0.57-1.89)**  **1.13 (0.63-2.01)** | **0.85 (0.59-1.22)**  **0.90 (0.55-1.49)**  **0.84 (0.58-1.22)**  **0.84 (0.58-1.22)** | **-**  **-**  **1.44 (0.32-7.80)**  **1.45 (0.32-7.66)** | **0.95 (0.79-1.14)**  **0.95 (0.79-1.14)**  **0.95 (0.78-1.15)**  **0.95 (0.78-1.14)** | **0.88 (0.57-1.37)**  **0.88 (0.57-1.37)**  **0.89 (0.65-1.22)**  **0.88 (0.65-1.21)** |
| **TCD** |  |  | **FE(IV)**  **RE(DL)**  **FE(BY)**  **FE(RA)** | **0.20 (0.01-4.07)**  **0.20 (0.01-4.07)**  **0.97 (0.69-1.36)**  **0.87 (0.62-1.25)** | **-**  **-**  **1.04 (0.73-1.49)**  **0.94 (0.65-1.37)** | **-**  **-**  **0.89 (0.57-1.40)**  **0.81 (0.51-1.28)** | **-**  **-**  **1.27 (0.67-2.47)**  **1.26 (0.68-2.32)** | **-**  **-**  **1.05 (0.65-1.67)**  **0.94 (0.58-1.52)** | **-**  **-**  **1.77 (0.38-9.78)**  **1.61 (0.35-8.79)** | **-**  **-**  **1.17 (0.83-1.66)**  **1.05 (0.74-1.52)** | **-**  **-**  **1.10 (0.73-1.66)**  **0.98 (0.65-1.51)** |
| **CLP** |  |  |  | **FE(IV)**  **RE(DL)**  **FE(BY)**  **FE(RA)** | **-**  **-**  **1.08 (0.82-1.42)**  **1.07 (0.82-1.41)** | **-**  **-**  **0.92 (0.63-1.36)**  **0.92 (0.63-1.36)** | **-**  **-**  **1.31 (0.71-2.48)**  **1.44 (0.78-2.63)** | **-**  **-**  **1.08 (0.71-1.63)**  **1.08 (0.71-1.62)** | **-**  **-**  **1.84 (0.41-10.1)**  **1.84 (0.41-9.9)** | **-**  **-**  **1.21 (0.93-1.57)**  **1.21 (0.93-1.57)** | **-**  **-**  **1.13 (0.79-1.64)**  **1.13 (0.79-1.62)** |
| **CLP**  **+ASA** |  |  |  |  | **FE(IV)**  **RE(DL)**  **FE(BY)**  **FE(RA)** | **0.86 (0.67-1.10)**  **0.86 (0.67-1.10)**  **0.86 (0.65-1.13)**  **0.86 (0.65-1.13)** | **-**  **-**  **1.22 (0.65-2.32)**  **1.34 (0.72-2.46)** | **-**  **-**  **1.00 (0.65-1.53)**  **1.00 (0.65-1.53)** | **-**  **-**  **1.70 (0.37-9.41)**  **1.72 (0.38-9.21)** | **-**  **-**  **1.13 (0.85-1.49)**  **1.13 (0.85-1.49)** | **-**  **-**  **1.05 (0.72-1.54)**  **1.05 (0.72-1.53)** |
| **TCG**  **+ASA** |  |  |  |  |  | **FE(IV)**  **RE(DL)**  **FE(BY)**  **FE(RA)** | **-**  **-**  **1.42 (0.72-2.87)**  **1.56 (0.79-3.04)** | **-**  **-**  **1.17 (0.70-1.93)**  **1.17 (0.71-1.93)** | **-**  **-**  **1.99 (0.43-11.2)**  **2.01 (0.43-11.0)** | **-**  **-**  **1.32 (0.88-1.94)**  **1.31 (0.89-1.94)** | **-**  **-**  **1.23 (0.77-1.97)**  **1.22 (0.77-1.96)** |
| **CSZ** |  |  |  |  |  |  | **FE(IV)**  **RE(DL)**  **FE(BY)**  **FE(RA)** | **-**  **-**  **0.82 (0.40-1.64)**  **0.75 (0.38-1.47)** | **-**  **-**  **1.39 (0.27-8.37)**  **1.26 (0.28-7.51)** | **-**  **-**  **0.92 (0.49-1.71)**  **0.84 (0.46-1.55)** | **-**  **-**  **0.87 (0.44-1.67)**  **0.78 (0.42-1.49)** |
| **CSZ**  **+ASA** |  |  |  |  |  |  |  | **FE(IV)**  **RE(DL)**  **FE(BY)**  **FE(RA)** | **1.64 (0.40-6.67)**  **1.64 (0.40-6.67)**  **1.71 (0.40-8.82)**  **1.72 (0.41-8.88)** | **-**  **-**  **1.12 (0.74-1.71)**  **1.12 (0.74-1.71)** | **-**  **-**  **1.05 (0.65-1.72)**  **1.05 (0.65-1.70)** |
| **TCD**  **+ASA** |  |  |  |  |  |  |  |  | **FE(IV)**  **RE(DL)**  **FE(BY)**  **FE(RA)** | **-**  **-**  **0.66 (0.12-2.99)**  **0.65 (0.12-2.95)** | **-**  **-**  **0.62 (0.11-2.86)**  **0.61 (0.11-2.81)** |
| **VXP**  **+ASA** |  |  |  |  |  |  |  |  |  | **FE(IV)**  **RE(DL)**  **FE(BY)**  **FE(RA)** | **-**  **-**  **0.94 (0.64-1.36)**  **0.93 (0.65-1.35)** |
| **PCD** |  |  |  |  |  |  |  |  |  |  | **FE(IV)**  **RE(DL)**  **FE(BY)**  **FE(RA)** |

| **SEVERE**  **BLEEDING** | **Placebo** | **ASA** | **TCD** | **CLP** | **CLP**  **+ASA** | **TCG**  **+ASA** | **CSZ** | **CSZ**  **+ASA** | **TCD**  **+ASA** | **VXP**  **+ASA** | **PCD** |
| --- | --- | --- | --- | --- | --- | --- | --- | --- | --- | --- | --- |
| **Placebo** | **FE(IV)**  **RE(DL)**  **FE(BY)**  **FE(RA)** | **1.06 (0.81-1.39)**  **1.06 (0.81-1.39)**  **1.14 (0.87-1.49)**  **-** | **2.12 (0.48-9.32)**  **2.12 (0.48-9.32)**  **5.03 (1.23-39.6)**  **-** | **-**  **-**  **1.01 (0.71-1.46)**  **-** | **-**  **-**  **1.48 (1.05-2.10)**  **-** | **-**  **-**  **1.22 (0.77-1.96)**  **-** | **1.45 (0.43-4.82)**  **1.45 (0.43-4.82)**  **2.61 (0.59-18.8)**  **-** | **-**  **-**  **0.94 (0.47-1.84)**  **-** | **-**  **-**  **1.52 (0.41-6.30)**  **-** | **-**  **-**  **1.80 (1.22-2.69)**  **-** | **1.00 (0.02-47.4)**  **1.00 (0.02-47.4)**  **0.75 (0.28-1.92)**  **-** |
| **ASA** |  | **FE(IV)**  **RE(DL)**  **FE(BY)**  **FE(RA)** | **-**  **-**  **4.44 (1.06-35.1)**  **-** | **0.88 (0.70-1.11)**  **0.88 (0.70-1.11)**  **0.89 (0.70-1.13)**  **-** | **1.29 (1.05-1.59)**  **1.36 (0.96-1.93)**  **1.30 (1.05-1.61)**  **-** | **-**  **-**  **1.08 (0.74-1.57)**  **-** | **0.35 (0.01-8.54)**  **0.35 (0.01-8.54)**  **2.29 (0.51-16.4)**  **-** | **0.83 (0.45-1.52)**  **0.83 (0.45-1.52)**  **0.82 (0.43-1.54)**  **-** | **-**  **-**  **1.32 (0.37-5.32)**  **-** | **1.58 (1.18-2.11)**  **1.58 (1.18-2.11)**  **1.58 (1.18-2.13)**  **-** | **0.67 (0.28-1.63)**  **0.67 (0.28-1.63)**  **0.66 (0.26-1.62)**  **-** |
| **TCD** |  |  | **FE(IV)**  **RE(DL)**  **FE(BY)**  **FE(RA)** | **0.33 (0.01-8.01)**  **0.33 (0.01-8.01)**  **0.20 (0.02-0.86)**  **-** | **-**  **-**  **0.29 (0.04-1.26)**  **-** | **-**  **-**  **0.24 (0.03-1.07)**  **-** | **-**  **-**  **0.51 (0.04-5.62)**  **-** | **-**  **-**  **0.18 (0.02-0.89)**  **-** | **-**  **-**  **0.30 (0.03-2.21)**  **-** | **-**  **-**  **0.35 (0.04-1.54)**  **-** | **-**  **-**  **0.14 (0.02-0.82)**  **-** |
| **CLP** |  |  |  | **FE(IV)**  **RE(DL)**  **FE(BY)**  **FE(RA)** | **-**  **-**  **1.46 (1.06-2.01)**  **-** | **-**  **-**  **1.21 (0.77-1.88)**  **-** | **-**  **-**  **2.57 (0.57-18.5)**  **-** | **-**  **-**  **0.92 (0.47-1.81)**  **-** | **-**  **-**  **1.49 (0.40-6.18)**  **-** | **-**  **-**  **1.78 (1.22-2.58)**  **-** | **-**  **-**  **0.74 (0.28-1.88)**  **-** |
| **CLP**  **+ASA** |  |  |  |  | **FE(IV)**  **RE(DL)**  **FE(BY)**  **FE(RA)** | **0.81 (0.61-1.08)**  **0.81 (0.61-1.08)**  **0.83 (0.61-1.13)**  **-** | **-**  **-**  **1.77 (0.39-12.9)**  **-** | **-**  **-**  **0.63 (0.32-1.23)**  **-** | **-**  **-**  **1.02 (0.28-4.17)**  **-** | **-**  **-**  **1.22 (0.85-1.74)**  **-** | **-**  **-**  **0.51 (0.19-1.29)**  **-** |
| **TCG**  **+ASA** |  |  |  |  |  | **FE(IV)**  **RE(DL)**  **FE(BY)**  **FE(RA)** | **-**  **-**  **2.13 (0.45-15.9)**  **-** | **-**  **-**  **0.76 (0.36-1.58)**  **-** | **-**  **-**  **1.23 (0.32-5.21)**  **-** | **-**  **-**  **1.47 (0.92-2.37)**  **-** | **-**  **-**  **0.61 (0.22-1.64)**  **-** |
| **CSZ** |  |  |  |  |  |  | **FE(IV)**  **RE(DL)**  **FE(BY)**  **FE(RA)** | **-**  **-**  **0.37 (0.05-1.86)**  **-** | **-**  **-**  **0.58 (0.06-4.58)**  **-** | **-**  **-**  **0.69 (0.10-3.23)**  **-** | **-**  **-**  **0.28 (0.03-1.69)**  **-** |
| **CSZ**  **+ASA** |  |  |  |  |  |  |  | **FE(IV)**  **RE(DL)**  **FE(BY)**  **FE(RA)** | **1.56 (0.52-4.76)**  **1.56 (0.52-4.76)**  **1.62 (0.53-5.55)**  **-** | **-**  **-**  **1.92 (0.96-3.89)**  **-** | **-**  **-**  **0.80 (0.26-2.43)**  **-** |
| **TCD**  **+ASA** |  |  |  |  |  |  |  |  | **FE(IV)**  **RE(DL)**  **FE(BY)**  **FE(RA)** | **-**  **-**  **1.20 (0.28-4.42)**  **-** | **-**  **-**  **0.49 (0.09-2.35)**  **-** |
| **VXP**  **+ASA** |  |  |  |  |  |  |  |  |  | **FE(IV)**  **RE(DL)**  **FE(BY)**  **FE(RA)** | **-**  **-**  **0.42 (0.16-1.08)**  **-** |
| **PCD** |  |  |  |  |  |  |  |  |  |  | **FE(IV)**  **RE(DL)**  **FE(BY)**  **FE(RA)** |

| **MAJOR**  **AMPUTATIONS** | **Placebo** | **ASA** | **TCD** | **CLP** | **CLP**  **+ASA** | **TCG**  **+ASA** | **CSZ** | **PCD** |
| --- | --- | --- | --- | --- | --- | --- | --- | --- |
| **Placebo** | **FE(IV)**  **RE(DL)**  **FE(BY)**  **FE(RA)** | **0.93 (0.59-1.47)**  **0.93 (0.59-1.47)**  **0.93 (0.59-1.46)**  **0.89 (0.57-1.44)** | **0.86 (0.31-2.37)**  **0.86 (0.31-2.37)**  **0.86 (0.29-2.46)**  **0.72 (0.23-2.65)** | **-**  **-**  **1.02 (0.56-1.89)**  **0.99 (0.54-1.83)** | **-**  **-**  **0.63 (0.35-1.15)**  **0.61 (0.33-1.13)** | **-**  **-**  **0.83 (0.30-2.33)**  **0.79 (0.28-2.28)** | **-**  **-**  **4.68 (0.41-173.5)**  **4.06 (0.35-178.2)** | **0.84 (0.26-2.73)**  **0.84 (0.26-2.73)**  **0.85 (0.32-2.32)**  **0.74 (0.26-2.28)** |
| **ASA** |  | **FE(IV)**  **RE(DL)**  **FE(BY)**  **FE(RA)** | **-**  **-**  **0.92 (0.29-2.92)**  **0.81 (0.25-2.97)** | **1.10 (0.74-1.63)**  **1.10 (0.74-1.63)**  **1.10 (0.74-1.64)**  **1.11 (0.74-1.65)** | **0.69 (0.47-1.00)**  **0.69 (0.47-1.00)**  **0.68 (0.46-0.99)**  **0.68 (0.46-1.00)** | **-**  **-**  **0.89 (0.36-2.28)**  **0.88 (0.36-2.27)** | **1.00 (0.06-15.8)**  **1.00 (0.06-15.8)**  **5.07 (0.43-188.0)**  **4.55 (0.39-203.7)** | **1.00 (0.25-4.01)**  **1.00 (0.25-4.01)**  **0.92 (0.33-2.55)**  **0.84 (0.29-2.41)** |
| **TCD** |  |  | **FE(IV)**  **RE(DL)**  **FE(BY)**  **FE(RA)** | **-**  **-**  **1.19 (0.35-4.07)**  **1.36 (0.35-4.79)** | **-**  **-**  **0.74 (0.22-2.50)**  **0.84 (0.22-2.90)** | **-**  **-**  **0.97 (0.22-4.28)**  **1.10 (0.22-4.90)** | **0.25 (0.03-2.17)**  **0.25 (0.03-2.17)**  **5.35 (0.64-177.3)**  **5.46 (0.68-205.9)** | **-**  **-**  **0.99 (0.24-4.25)**  **1.03 (0.24-4.22)** |
| **CLP** |  |  |  | **FE(IV)**  **RE(DL)**  **FE(BY)**  **FE(RA)** | **-**  **-**  **0.62 (0.36-1.08)**  **0.62 (0.35-1.07)** | **-**  **-**  **0.81 (0.30-2.23)**  **0.80 (0.30-2.22)** | **-**  **-**  **4.61 (0.37-170.6)**  **4.13 (0.33-186.9)** | **-**  **-**  **0.83 (0.28-2.49)**  **0.76 (0.25-2.36)** |
| **CLP**  **+ASA** |  |  |  |  | **FE(IV)**  **RE(DL)**  **FE(BY)**  **FE(RA)** | **1.29 (0.57-2.95)**  **1.29 (0.57-2.95)**  **1.30 (0.57-3.08)**  **1.30 (0.57-3.06)** | **-**  **-**  **7.41 (0.61-276.6)**  **6.69 (0.55-302.9)** | **-**  **-**  **1.35 (0.45-3.98)**  **1.23 (0.40-3.79)** |
| **TCG**  **+ASA** |  |  |  |  |  | **FE(IV)**  **RE(DL)**  **FE(BY)**  **FE(RA)** | **-**  **-**  **5.70 (0.41-225.2)**  **5.20 (0.37-252.5)** | **-**  **-**  **1.03 (0.26-4.04)**  **0.94 (0.23-3.84)** |
| **CSZ** |  |  |  |  |  |  | **FE(IV)**  **RE(DL)**  **FE(BY)**  **FE(RA)** | **-**  **-**  **0.18 (0.004-2.57)**  **0.18 (0.004-2.39)** |
| **PCD** |  |  |  |  |  |  |  | **FE(IV)**  **RE(DL)**  **FE(BY)**  **FE(RA)** |

| **CARDIOVASCULAR**  **DEATHS** | **Placebo** | **ASA** | **TCD** | **CLP** | **CLP**  **+ASA** | **TCG**  **+ASA** | **CSZ**  **+ASA** | **TCD**  **+ASA** | **VXP**  **+ASA** | **PCD** |
| --- | --- | --- | --- | --- | --- | --- | --- | --- | --- | --- |
| **Placebo** | **FE(IV)**  **RE(DL)**  **FE(BY)**  **FE(RA)** | **1.00 (0.80-1.25)**  **1.00 (0.80-1.25)**  **1.05 (0.84-1.30)**  **0.99 (0.81-1.24)** | **0.61 (0.41-0.92)**  **0.61 (0.41-0.92)**  **0.59 (0.38-0.89)**  **0.63 (0.41-0.92)** | **-**  **-**  **0.82 (0.58-1.16)**  **0.78 (0.56-1.10)** | **-**  **-**  **0.97 (0.67-1.40)**  **0.93 (0.65-1.35)** | **-**  **-**  **0.72 (0.41-1.25)**  **0.69 (0.40-1.19)** | **-**  **-**  **0.90 (0.54-1.53)**  **0.86 (0.50-1.46)** | **-**  **-**  **1.57 (0.34-8.99)**  **1.49 (0.31-8.46)** | **-**  **-**  **0.94 (0.65-1.35)**  **0.89 (0.62-1.28)** | **0.84 (0.44-1.63)**  **0.84 (0.44-1.63)**  **0.69 (0.41-1.13)**  **0.65 (0.39-1.06)** |
| **ASA** |  | **FE(IV)**  **RE(DL)**  **FE(BY)**  **FE(RA)** | **-**  **-**  **0.56 (0.35-0.90)**  **0.63 (0.39-0.98)** | **0.78 (0.60-1.02)**  **0.78 (0.60-1.02)**  **0.78 (0.60-1.03)**  **0.79 (0.60-1.03)** | **0.93 (0.69-1.25)**  **0.93 (0.69-1.25)**  **0.92 (0.69-1.25)**  **0.93 (0.69-1.26)** | **-**  **-**  **0.68 (0.41-1.14)**  **0.69 (0.41-1.14)** | **0.86 (0.54-1.39)**  **0.86 (0.54-1.39)**  **0.86 (0.53-1.40)**  **0.86 (0.53-1.40)** | **-**  **-**  **1.51 (0.32-8.59)**  **1.49 (0.32-8.35)** | **0.90 (0.68-1.20)**  **0.90 (0.68-1.20)**  **0.90 (0.67-1.20)**  **0.90 (0.67-1.20)** | **0.55 (0.27-1.10)**  **0.55 (0.27-1.10)**  **0.66 (0.40-1.08)**  **0.65 (0.40-1.05)** |
| **TCD** |  |  | **FE(IV)**  **RE(DL)**  **FE(BY)**  **FE(RA)** | **0.33 (0.01-8.00)**  **0.33 (0.01-8.00)**  **1.40 (0.81-2.43)**  **1.26 (0.75-2.16)** | **-**  **-**  **1.65 (0.95-2.91)**  **1.49 (0.87-2.60)** | **-**  **-**  **1.22 (0.61-2.44)**  **1.10 (0.56-2.20)** | **-**  **-**  **1.54 (0.79-3.01)**  **1.39 (0.71-2.72)** | **-**  **-**  **2.69 (0.54-15.9)**  **2.40 (0.48-14.2)** | **-**  **-**  **1.60 (0.92-2.82)**  **1.44 (0.84-2.49)** | **-**  **-**  **1.18 (0.61-2.27)**  **1.04 (0.55-1.98)** |
| **CLP** |  |  |  | **FE(IV)**  **RE(DL)**  **FE(BY)**  **FE(RA)** | **-**  **-**  **1.18 (0.79-1.77)**  **1.19 (0.80-1.77)** | **-**  **-**  **0.87 (0.49-1.54)**  **0.87 (0.49-1.55)** | **-**  **-**  **1.10 (0.63-1.91)**  **1.11 (0.63-1.93)** | **-**  **-**  **1.93 (0.40-11.2)**  **1.90 (0.40-10.9)** | **-**  **-**  **1.15 (0.77-1.70)**  **1.15 (0.77-1.69)** | **-**  **-**  **0.84 (0.47-1.46)**  **0.83 (0.47-1.44)** |
| **CLP**  **+ASA** |  |  |  |  | **FE(IV)**  **RE(DL)**  **FE(BY)**  **FE(RA)** | **0.71 (0.43-1.18)**  **0.71 (0.43-1.18)**  **0.74 (0.49-1.11)**  **0.74 (0.48-1.11)** | **-**  **-**  **0.93 (0.53-1.64)**  **0.93 (0.53-1.63)** | **-**  **-**  **1.64 (0.34-9.54)**  **1.60 (0.33-9.25)** | **-**  **-**  **0.97 (0.64-1.48)**  **0.97 (0.64-1.46)** | **-**  **-**  **0.71 (0.39-1.27)**  **0.70 (0.39-1.23)** |
| **TCG**  **+ASA** |  |  |  |  |  | **FE(IV)**  **RE(DL)**  **FE(BY)**  **FE(RA)** | **-**  **-**  **1.27 (0.63-2.54)**  **1.26 (0.63-2.52)** | **-**  **-**  **2.22 (0.43-13.5)**  **2.19 (0.43-12.9)** | **-**  **-**  **1.32 (0.73-2.39)**  **1.31 (0.73-2.34)** | **-**  **-**  **0.97 (0.47-1.97)**  **0.95 (0.47-1.90)** |
| **CSZ**  **+ASA** |  |  |  |  |  |  | **FE(IV)**  **RE(DL)**  **FE(BY)**  **FE(RA)** | **0.61 (0.15-2.52)**  **0.61 (0.15-2.52)**  **1.74 (0.41-9.22)**  **1.72 (0.40-9.05)** | **-**  **-**  **1.04 (0.59-1.82)**  **1.04 (0.59-1.83)** | **-**  **-**  **0.76 (0.38-1.52)**  **0.75 (0.38-1.50)** |
| **TCD**  **+ASA** |  |  |  |  |  |  |  | **FE(IV)**  **RE(DL)**  **FE(BY)**  **FE(RA)** | **-**  **-**  **0.60 (0.10-2.85)**  **0.60 (0.11-2.89)** | **-**  **-**  **0.44 (0.07-2.18)**  **0.44 (0.07-2.19)** |
| **VXP**  **+ASA** |  |  |  |  |  |  |  |  | **FE(IV)**  **RE(DL)**  **FE(BY)**  **FE(RA)** | **-**  **-**  **0.73 (0.41-1.30)**  **0.72 (0.41-1.27)** |
| **PCD** |  |  |  |  |  |  |  |  |  | **FE(IV)**  **RE(DL)**  **FE(BY)**  **FE(RA)** |

| **NON-FATAL STROKE** | **Placebo** | **ASA** | **TCD** | **CLP** | **CLP**  **+ASA** | **TCG**  **+ASA** | **CSZ** | **CSZ**  **+ASA** | **VXP**  **+ASA** | **PCD** |
| --- | --- | --- | --- | --- | --- | --- | --- | --- | --- | --- |
| **Placebo** | **FE(IV)**  **RE(DL)**  **FE(BY)**  **FE(RA)** | **0.78 (0.59-1.03)**  **0.78 (0.59-1.03)**  **0.73 (0.55-0.97)**  **0.49 (0.29-0.72)** | **0.80 (0.46-1.39)**  **0.80 (0.46-1.39)**  **0.74 (0.41-1.30)**  **0.74 (0.45-1.15)** | **-**  **-**  **0.69 (0.45-1.07)**  **0.46 (0.24-0.78)** | **-**  **-**  **0.66 (0.40-1.08)**  **0.43 (0.22-0.78)** | **-**  **-**  **0.93 (0.37-2.42)**  **0.60 (0.22-1.67)** | **0.46 (0.15-1.37)**  **0.46 (0.15-1.37)**  **0.71 (0.23-2.29)**  **0.79 (0.29-1.84)** | **-**  **-**  **0.45 (0.19-1.05)**  **0.29 (0.11-0.73)** | **-**  **-**  **0.66 (0.40-1.08)**  **0.43 (0.22-0.78)** | **0.90 (0.37-2.21)**  **0.90 (0.37-2.21)**  **0.88 (0.33-2.37)**  **0.48 (0.19-1.16)** |
| **ASA** |  | **FE(IV)**  **RE(DL)**  **FE(BY)**  **FE(RA)** | **-**  **-**  **1.01 (0.53-1.89)**  **1.56 (0.82-2.85)** | **0.95 (0.68-1.31)**  **0.95 (0.68-1.31)**  **0.94 (0.68-1.31)**  **0.95 (0.68-1.32)** | **0.90 (0.60-1.35)**  **0.90 (0.60-1.35)**  **0.90 (0.60-1.36)**  **0.90 (0.60-1.36)** | **-**  **-**  **1.27 (0.52-3.18)**  **1.26 (0.52-3.16)** | **7.34 (0.38-141.4)**  **7.34 (0.38-141.4)**  **0.98 (0.31-3.20)**  **1.67 (0.54-4.63)** | **0.57 (0.25-1.30)**  **0.77 (0.16-3.63)**  **0.62 (0.27-1.37)**  **0.62 (0.27-1.37)** | **0.90 (0.60-1.35)**  **0.90 (0.60-1.35)**  **0.90 (0.59-1.36)**  **0.90 (0.59-1.36)** | **2.01 (0.81-4.96)**  **2.01 (0.81-4.96)**  **1.20 (0.43-3.35)**  **1.04 (0.43-2.18)** |
| **TCD** |  |  | **FE(IV)**  **RE(DL)**  **FE(BY)**  **FE(RA)** | **-**  **-**  **0.93 (0.46-1.93)**  **0.61 (0.31-1.26)** | **-**  **-**  **0.89 (0.42-1.90)**  **0.58 (0.28-1.25)** | **-**  **-**  **1.26 (0.44-3.82)**  **0.81 (0.28-2.46)** | **-**  **-**  **0.97 (0.28-3.53)**  **1.08 (0.36-2.86)** | **-**  **-**  **0.62 (0.22-1.71)**  **0.40 (0.14-1.10)** | **-**  **-**  **0.89 (0.42-1.91)**  **0.58 (0.27-1.24)** | **-**  **-**  **1.19 (0.38-3.74)**  **0.66 (0.24-1.76)** |
| **CLP** |  |  |  | **FE(IV)**  **RE(DL)**  **FE(BY)**  **FE(RA)** | **-**  **-**  **0.95 (0.56-1.62)**  **0.95 (0.56-1.62)** | **-**  **-**  **1.35 (0.52-3.57)**  **1.33 (0.51-3.53)** | **-**  **-**  **1.03 (0.31-3.57)**  **1.76 (0.55-5.11)** | **-**  **-**  **0.66 (0.27-1.54)**  **0.66 (0.27-1.56)** | **-**  **-**  **0.96 (0.56-1.62)**  **0.95 (0.56-1.61)** | **-**  **-**  **1.28 (0.43-3.74)**  **1.10 (0.42-2.48)** |
| **CLP**  **+ASA** |  |  |  |  | **FE(IV)**  **RE(DL)**  **FE(BY)**  **FE(RA)** | **1.36 (0.63-2.94)**  **1.36 (0.63-2.94)**  **1.41 (0.64-3.20)**  **1.40 (0.64-3.15)** | **-**  **-**  **1.08 (0.32-3.81)**  **1.85 (0.56-5.53)** | **-**  **-**  **0.69 (0.28-1.67)**  **0.69 (0.27-1.68)** | **-**  **-**  **0.99 (0.55-1.79)**  **1.00 (0.56-1.79)** | **-**  **-**  **1.34 (0.44-4.04)**  **1.15 (0.44-2.69)** |
| **TCG**  **+ASA** |  |  |  |  |  | **FE(IV)**  **RE(DL)**  **FE(BY)**  **FE(RA)** | **-**  **-**  **0.77 (0.17-3.40)**  **1.31 (0.32-5.06)** | **-**  **-**  **0.49 (0.14-1.58)**  **0.49 (0.14-1.63)** | **-**  **-**  **0.71 (0.26-1.91)**  **0.71 (0.26-1.89)** | **-**  **-**  **0.95 (0.24-3.70)**  **0.82 (0.23-2.61)** |
| **CSZ**  **+ASA** |  |  |  |  |  |  | **FE(IV)**  **RE(DL)**  **FE(BY)**  **FE(RA)** | **-**  **-**  **0.64 (0.15-2.56)**  **0.37 (0.10-1.45)** | **-**  **-**  **0.92 (0.26-3.15)**  **0.54 (0.18-1.76)** | **-**  **-**  **1.23 (0.27-5.44)**  **0.61 (0.17-2.36)** |
| **TCD**  **+ASA** |  |  |  |  |  |  |  | **FE(IV)**  **RE(DL)**  **FE(BY)**  **FE(RA)** | **-**  **-**  **1.44 (0.59-3.61)**  **1.45 (0.59-3.64)** | **-**  **-**  **1.93 (0.53-7.22)**  **1.66 (0.51-5.14)** |
| **VXP**  **+ASA** |  |  |  |  |  |  |  |  | **FE(IV)**  **RE(DL)**  **FE(BY)**  **FE(RA)** | **-**  **-**  **1.34 (0.44-4.02)**  **1.15 (0.44-2.71)** |
| **PCD** |  |  |  |  |  |  |  |  |  | **FE(IV)**  **RE(DL)**  **FE(BY)**  **FE(RA)** |

| **NON-FATAL**  **MI** | **Placebo** | **ASA** | **TCD** | **CLP** | **CLP**  **+ASA** | **TCG**  **+ASA** | **CSZ** | **CSZ**  **+ASA** | **VXP**  **+ASA** | **PCD** |
| --- | --- | --- | --- | --- | --- | --- | --- | --- | --- | --- |
| **Placebo** | **FE(IV)**  **RE(DL)**  **FE(BY)**  **FE(RA)** | **0.98 (0.78-1.23)**  **0.98 (0.78-1.23)**  **0.96 (0.78-1.19)**  **0.81 (0.60-1.16)** | **0.99 (0.67-1.49)**  **0.99 (0.67-1.49)**  **0.94 (0.62-1.41)**  **0.95 (0.65-1.38)** | **-**  **-**  **0.60 (0.40-0.91)**  **0.51 (0.32-0.84)** | **-**  **-**  **0.61 (0.40-0.91)**  **0.52 (0.32-0.83)** | **-**  **-**  **0.51 (0.30-0.87)**  **0.43 (0.24-0.78)** | **1.11 (0.53-2.31)**  **1.11 (0.53-2.31)**  **1.05 (0.52-2.14)**  **1.21 (0.60-2.33)** | **-**  **-**  **1.00 (0.10-10.8)**  **0.84 (0.09-0.84)** | **-**  **-**  **0.95 (0.67-1.36)**  **0.81 (0.53-1.26)** | **0.78 (0.38-1.58)**  **0.78 (0.38-1.58)**  **0.78 (0.44-1.36)**  **0.67 (0.38-1.21)** |
| **ASA** |  | **FE(IV)**  **RE(DL)**  **FE(BY)**  **FE(RA)** | **-**  **-**  **0.98 (0.62-1.55)**  **1.17 (0.68-1.88)** | **0.62 (0.46-0.88)**  **0.62 (0.46-0.88)**  **0.63 (0.44-0.89)**  **0.63 (0.44-0.89)** | **0.64 (0.46-0.89)**  **0.64 (0.46-0.89)**  **0.63 (0.45-0.89)**  **0.63 (0.45-0.88)** | **-**  **-**  **0.53 (0.32-0.87)**  **0.53 (0.32-0.85)** | **0.52 (0.05-5.75)**  **0.52 (0.05-5.75)**  **1.08 (0.53-2.28)**  **1.49 (0.62-3.23)** | **1.05 (0.15-7.35)**  **1.05 (0.15-7.35)**  **1.03 (0.11-11.2)**  **1.04 (0.11-9.55)** | **0.99 (0.75-1.30)**  **0.99 (0.75-1.30)**  **0.99 (0.75-1.31)**  **0.99 (0.75-1.31)** | **0.84 (0.36-1.93)**  **0.84 (0.36-1.93)**  **0.81 (0.45-1.42)**  **0.82 (0.47-1.39)** |
| **TCD** |  |  | **FE(IV)**  **RE(DL)**  **FE(BY)**  **FE(RA)** | **0.86 (0.76-0.97)**  **0.86 (0.76-0.97)**  **0.64 (0.36-1.14)**  **0.54 (0.30-1.02)** | **-**  **-**  **0.65 (0.36-1.15)**  **0.54 (0.30-1.01)** | **-**  **-**  **0.54 (0.27-1.06)**  **0.45 (0.23-0.93)** | **-**  **-**  **1.11 (0.50-2.56)**  **1.27 (0.57-2.70)** | **-**  **-**  **1.06 (0.11-11.7)**  **0.89 (0.09-8.62)** | **-**  **-**  **1.02 (0.59-1.73)**  **0.85 (0.49-1.56)** | **-**  **-**  **0.83 (0.41-1.65)**  **0.70 (0.35-1.46)** |
| **CLP** |  |  |  | **FE(IV)**  **RE(DL)**  **FE(BY)**  **FE(RA)** | **-**  **-**  **1.01 (0.62-1.64)**  **0.99 (0.62-1.63)** | **-**  **-**  **0.84 (0.46-1.53)**  **0.84 (0.46-1.53)** | **-**  **-**  **1.73 (0.77-3.95)**  **2.36 (0.92-5.58)** | **-**  **-**  **1.65 (0.17-17.9)**  **1.64 (0.17-15.8)** | **-**  **-**  **1.58 (1.01-2.47)**  **1.58 (1.01-2.47)** | **-**  **-**  **1.29 (0.65-2.49)**  **1.31 (0.68-2.49)** |
| **CLP**  **+ASA** |  |  |  |  | **FE(IV)**  **RE(DL)**  **FE(BY)**  **FE(RA)** | **0.84 (0.60-1.18)**  **0.84 (0.60-1.18)**  **0.84 (0.58-1.20)**  **0.84 (0.58-1.20)** | **-**  **-**  **1.72 (0.77-3.90)**  **2.36 (0.93-5.52)** | **-**  **-**  **1.64 (0.17-18.3)**  **1.65 (0.17-15.4)** | **-**  **-**  **1.57 (1.01-2.44)**  **1.58 (1.02-2.45)** | **-**  **-**  **1.28 (0.66-2.48)**  **1.31 (0.69-2.45)** |
| **TCG**  **+ASA** |  |  |  |  |  | **FE(IV)**  **RE(DL)**  **FE(BY)**  **FE(RA)** | **-**  **-**  **2.06 (0.86-5.04)**  **2.81 (1.04-7.08)** | **-**  **-**  **1.97 (0.20-22.3)**  **1.97 (0.20-18.9)** | **-**  **-**  **1.88 (1.06-3.30)**  **1.89 (1.08-3.30)** | **-**  **-**  **1.53 (0.72-3.25)**  **1.56 (0.75-3.23)** |
| **CSZ**  **+ASA** |  |  |  |  |  |  | **FE(IV)**  **RE(DL)**  **FE(BY)**  **FE(RA)** | **-**  **-**  **0.95 (0.09-11.7)**  **0.70 (0.06-7.67)** | **-**  **-**  **0.91 (0.41-1.98)**  **0.67 (0.29-1.67)** | **-**  **-**  **0.74 (0.30-1.80)**  **0.55 (0.22-1.51)** |
| **TCD**  **+ASA** |  |  |  |  |  |  |  | **FE(IV)**  **RE(DL)**  **FE(BY)**  **FE(RA)** | **-**  **-**  **0.96 (0.09-9.11)**  **0.95 (0.10-9.33)** | **-**  **-**  **0.78 (0.07-7.76)**  **0.79 (0.08-8.10)** |
| **VXP**  **+ASA** |  |  |  |  |  |  |  |  | **FE(IV)**  **RE(DL)**  **FE(BY)**  **FE(RA)** | **-**  **-**  **0.81 (0.42-1.52)**  **0.83 (0.45-1.51)** |
| **PCD** |  |  |  |  |  |  |  |  |  | **FE(IV)**  **RE(DL)**  **FE(BY)**  **FE(RA)** |

**APPENDIX 7. Model fit, regression and consistency analysis**

| **Table 1. Model fit and parsimony** | | | |
| --- | --- | --- | --- |
| **Endpoint** | **Model** | **DIC** | **Residual deviance** |
| Composite endpoint  (98 study arms) | Standard Fixed Effects | 486.584 | 92.85 |
|  | Baseline Risk-adjusted | 489.181 | 94.25 |
| Cardiovascular deaths  (80 study arms) | Standard Fixed Effects | 354.058 | 71.12 |
|  | Baseline Risk-adjusted | 355.716 | 71.66 |
| Non-fatal MI  (88 study arms) | Standard Fixed Effects | 343.891 | 80.02 |
|  | Baseline Risk-adjusted | 348.876 | 83.46 |
| Non-fatal Stroke  (86 study arms) | Standard Fixed Effects | 305.327 | 87.28 |
|  | Baseline Risk-adjusted | 262.571 | 90.59 |
| Major amputations  (22 study arms) | Standard Fixed Effects | 124.624 | 20.13 |
|  | Baseline Risk-adjusted | 126.100 | 20.82 |
| Severe bleeding  (84 study arms) | Standard Fixed Effects | 310.188 | 79.76 |
|  | Baseline Risk-adjusted | n/a | n/a |

DIC was similar in Standard Fixed effects and Baseline Risk Adjusted models in most cases; significantly less though in case of non-fatal stroke. In addition, Baseline Risk Adjusted models generally approximated better the total number of study arms analysed in each case. Hence, models with baseline-risk adjustment are considered to produce a slightly better model fit than the standard unadjusted ones for the present network meta-analysis.

| **Table 2. Baseline risk meta-regression analysis (log scale)** | | |
| --- | --- | --- |
| **Endpoint** | **Regression coefficient** | **95% CrI** |
| Composite endpoint | -0.1266 | (-0.2622) – 0.0318 |
| Cardiovascular deaths | -0.1679 | (-0.3190) – 0.0068 |
| Non-fatal MI | -0.1861 | (-0.4623) – 0.1433 |
| Non-fatal Stroke | -0.5940 | (-0.9984) – (-0.2355) |
| Major amputations | -0.0910 | (-0.3364) – 0.2345 |

| **Table 3. Network consistency analysis (log scale)** | | | |
| --- | --- | --- | --- |
| **Endpoint** | **Closed loop** | **Inconsistency** | **P value** |
| Composite endpoint | Cilostazol-Aspirin-Placebo | 0.83 ± 0.93 | 0.38 |
|  | Picotamide-Aspirin-Placebo | (-0.02) ± 0.32 | 0.94 |
| Cardiovascular deaths | Cilostazol-Aspirin-Placebo | n/a | n/a |
|  | Picotamide-Aspirin-Placebo | 0.44 ± 0.51 | 0.38 |
| Non-fatal MI | Cilostazol-Aspirin-Placebo | 0.99 ± 1.58 | 0.53 |
|  | Picotamide-Aspirin-Placebo | 0.07 ± 0.58 | 0.91 |
| Non-fatal Stroke | Cilostazol-Aspirin-Placebo | (-4.42) ± 1.63 | 0.007 |
|  | Picotamide-Aspirin-Placebo | 3.16 ± 1.07 | 0.003 |
| Major amputations | Cilostazol-Aspirin-Placebo | n/a | n/a |
|  | Picotamide-Aspirin-Placebo | 0.18 ± 1.04 | 0.87 |
| Severe bleeding | Cilostazol-Aspirin-Placebo | (-3.03) ± 2.07 | 0.14 |
|  | Picotamide-Aspirin-Placebo | (-0.05) ± 3.26 | 0.98 |

**WinBUGS Code - Poisson likelihood, log link, fixed effects**

model{ # *** PROGRAM STARTS

for(i in 1:ns){ # LOOP THROUGH STUDIES

mu[i] ~ dnorm(0,.0001) # vague priors for all trial baselines

for (k in 1:na[i]) { # LOOP THROUGH ARMS

r[i,k] ~ dpois(theta[i,k]) # Poisson likelihood

theta[i,k] <- lambda[i,k]*E[i,k] # failure rate * exposure

log(lambda[i,k]) <- mu[i] + d[t[i,k]] - d[t[i,1]] +(beta[t[i,k]]-beta[t[i,1]]) * (mu[i]-mx) # model for linear predictor

#Deviance contribution

dev[i,k] <- 2*((theta[i,k]-r[i,k]) + r[i,k]*log(r[i,k]/theta[i,k])) }

# summed residual deviance contribution for this trial

resdev[i] <- sum(dev[i,1:na[i]]) }

totresdev <- sum(resdev[]) # Total Residual Deviance

d[1]<-0 # treatment effect is zero for reference treatment

beta[1] <- 0 # covariate effect is zero for reference treatment

# vague priors for treatment effects

for (k in 2:nt){ d[k] ~ dnorm(0,.0001)

beta[k] <- B }

B ~ dnorm(0,.0001) # vague prior for covariate effect

# Provide estimates of treatment effects T[k] on the natural (probability) scale

# Given a Mean Effect, meanA, for 'standard' treatment A,

# with precision (1/variance) precA

A ~ dnorm(meanA,precA)

for (k in 1:nt) { log(T[k]) <- A + d[k] }

# pairwise RRs and bayesian p values for all possible pair-wise comparisons, if nt>2

for (c in 1:(nt-1)) {

for (k in (c+1):nt) {

or[c,k] <- exp(d[k] - d[c])

lor[c,k] <- (d[k]-d[c])

RD[c,k] <- T[k] - T[c]

RR[c,k] <- T[k]/T[c]

Pabove[c,k] <- step(lor[c,k])

Pbelow[c,k] <- 1-step(lor[c,k])

P[c,k] <- 2*min(Pabove[c,k], Pbelow[c,k]) } }

# ranking on relative scale

for (k in 1:nt) {

#rk[k] <- nt+1-rank(d[],k) # assumes events are good

rk[k] <- rank(d[],k) # assumes events are bad

best[k] <- equals(rk[k],1) #calculate probability that treat k is best

for (h in 1:nt){ prob[h,k] <- equals(rk[k],h) }} # calculates probability that treat k is h-th best

} # *** PROGRAM ENDS

**REFERENCES**

1. Thompson PD, Zimet R, Forbes WP, Zhang P (2002) Meta-analysis of results from eight randomized, placebo-controlled trials on the effect of cilostazol on patients with intermittent claudication. Am J Cardiol 90: 1314-1319.

2. (2002) Collaborative meta-analysis of randomised trials of antiplatelet therapy for prevention of death, myocardial infarction, and stroke in high risk patients. BMJ 324: 71-86.

3. Baigent C, Blackwell L, Collins R, Emberson J, Godwin J, et al. (2009) Aspirin in the primary and secondary prevention of vascular disease: collaborative meta-analysis of individual participant data from randomised trials. Lancet 373: 1849-1860.

4. Robless P, Mikhailidis DP, Stansby G (2001) Systematic review of antiplatelet therapy for the prevention of myocardial infarction, stroke or vascular death in patients with peripheral vascular disease. Br J Surg 88: 787-800.

5. Warner CJ, Greaves SW, Larson RJ, Stone DH, Powell RJ, et al. (2014) Cilostazol is associated with improved outcomes after peripheral endovascular interventions. J Vasc Surg 59: 1607-1614.

6. Berger JS, Krantz MJ, Kittelson JM, Hiatt WR (2009) Aspirin for the prevention of cardiovascular events in patients with peripheral artery disease: a meta-analysis of randomized trials. JAMA 301: 1909-1919.

7. Uchiyama S, Demaerschalk BM, Goto S, Shinohara Y, Gotoh F, et al. (2009) Stroke prevention by cilostazol in patients with atherothrombosis: meta-analysis of placebo-controlled randomized trials. J Stroke Cerebrovasc Dis 18: 482-490.

8. Moher D, Liberati A, Tetzlaff J, Altman DG (2009) Preferred reporting items for systematic reviews and meta-analyses: the PRISMA statement. BMJ 339: b2535.

9. Jadad AR, Moore RA, Carroll D, Jenkinson C, Reynolds DJ, et al. (1996) Assessing the quality of reports of randomized clinical trials: is blinding necessary? Control Clin Trials 17: 1-12.

10. Dias S, Welton NJ, Sutton AJ, Caldwell DM, Guobing L, et al. (2011) NICE DSU technical support document 4: Inconsistency in networks of evidence based on randomised controlled trials. Last updated April 2012: Available from <http://www.nicedsu.org.uk>.

11. Dias S, Welton NJ, Sutton AJ, Ades AE (2011) NICE DSU technical support document 2: A generalised linear modelling framework for pairwise and network meta-analysis of randomized controlled trials. Last updated March 2013: Available from <http://www.nicedsu.org.uk>.

12. Dias S, Sutton AJ, Welton NJ, Ades AE (2011) NICE DSU technical support document 3: Heterogeneity: Subgroups, Meta-regression, bias and bias adjustment. Last updated April 2012: Available from <http://www.nicedsu.org.uk>.

13. Dias S, Sutton AJ, Ades AE, Welton NJ (2013) Evidence synthesis for decision making 2: a generalized linear modeling framework for pairwise and network meta-analysis of randomized controlled trials. Med Decis Making 33: 607-617.

14. Salanti G, Ades AE, Ioannidis JP (2011) Graphical methods and numerical summaries for presenting results from multiple-treatment meta-analysis: an overview and tutorial. J Clin Epidemiol 64: 163-171.

15. Dias S, Sutton AJ, Ades AE, Welton NJ (2012) A Generalized Linear Modeling Framework for Pairwise and Network Meta-analysis of Randomized Controlled Trials. Med Decis Making.

16. Friedrich JO, Adhikari NK, Beyene J (2007) Inclusion of zero total event trials in meta-analyses maintains analytic consistency and incorporates all available data. BMC Med Res Methodol 7: 5.

17. Thompson SG, Smith TC, Sharp SJ (1997) Investigating underlying risk as a source of heterogeneity in meta-analysis. Stat Med 16: 2741-2758.

18. Achana FA, Cooper NJ, Dias S, Lu G, Rice SJ, et al. (2013) Extending methods for investigating the relationship between treatment effect and baseline risk from pairwise meta-analysis to network meta-analysis. Stat Med 32: 752-771.

19. Dias S, Sutton AJ, Welton NJ, Ades AE (2013) Evidence synthesis for decision making 3: heterogeneity--subgroups, meta-regression, bias, and bias-adjustment. Med Decis Making 33: 618-640.

20. Brooks SP, Gelman A (1998) General Methods for Monitoring Convergence of Iterative Simulations. Journal of Computational and Graphical Statistics 7: 434-455.

21. Mills EJ, Thorlund K, Ioannidis JP (2013) Demystifying trial networks and network meta-analysis. BMJ 346: f2914.

22. Jansen JP, Naci H (2013) Is network meta-analysis as valid as standard pairwise meta-analysis? It all depends on the distribution of effect modifiers. BMC Med 11: 159.

23. Higgins JP, Thompson SG, Deeks JJ, Altman DG (2003) Measuring inconsistency in meta-analyses. BMJ 327: 557-560.

24. Egger M, Davey Smith G, Schneider M, Minder C (1997) Bias in meta-analysis detected by a simple, graphical test. BMJ 315: 629-634.

25. Dias S, Welton NJ, Caldwell DM, Ades AE (2010) Checking consistency in mixed treatment comparison meta-analysis. Stat Med 29: 932-944.

26. Bucher HC, Guyatt GH, Griffith LE, Walter SD (1997) The results of direct and indirect treatment comparisons in meta-analysis of randomized controlled trials. J Clin Epidemiol 50: 683-691.

27. Belch J, MacCuish A, Campbell I, Cobbe S, Taylor R, et al. (2008) The prevention of progression of arterial disease and diabetes (POPADAD) trial: factorial randomised placebo controlled trial of aspirin and antioxidants in patients with diabetes and asymptomatic peripheral arterial disease. BMJ 337: a1840.

28. Fowkes FG, Price JF, Stewart MC, Butcher I, Leng GC, et al. (2010) Aspirin for prevention of cardiovascular events in a general population screened for a low ankle brachial index: a randomized controlled trial. JAMA 303: 841-848.

29. Greenland S, Poole C (2013) Living with p values: resurrecting a Bayesian perspective on frequentist statistics. Epidemiology 24: 62-68.

30. Hess H, Keil-Kuri E (1975) Theoretische grundlagen der prophylaxe obliterierender arteriopathien mit aggregationshemmern und ergebnisse einer langzeitstudie mit ASS (Colfarit). In: Marx R, Breddin HK (Eds) Proceedings of Colfarit Symposium III 80-87.

31. Ehresmann U, Alemany J, Loew D (1977) Prophylaxe von rezidivverschlüssen nach revaskularisationseingriffen mit acetylsalicylsäure. Med Welt 28: 1157-1162

32. Green RM, Roedersheimer LR, DeWeese JA (1982) Effects of aspirin and dipyridamole on expanded polytetrafluoroethylene graft patency. Surgery 92: 1016-1026.

33. Schoop W, Levy H (1983) Prevention of peripheral arterial occlusive disease with antiaggregants. Thromb Haemost 50: (Abstract 0416).

34. Schoop W, Levy H, Schoop B, Gaentzsch A (1983) Experimentelle und klinische studien zu der sekundären prävention der peripheren arteriosklerose. . In: Bollinger A , Rhyner K (Eds) Thrombozytenfunktionshemmer, wirkungsmechanismen,dosierung und praktische Stuttgart: Thieme 49-58.

35. Schoop W (1984) Spätergebnisse bei konservativer therapie der arteriellen verschlußkrankheit. Der Internist 25: 429-433.

36. Kohler TR, Kaufman JL, Kacoyanis G, Clowes A, Donaldson MC, et al. (1984) Effect of aspirin and dipyridamole on the patency of lower extremity bypass grafts. Surgery 96: 462-466.

37. Goldman MR, McCollum C (1984) A prospective randomised study to examine the effect of aspirin plus dipyridamole on the patency of prosthetic femoro-popliteal grafts. Vasc Surg 18: 217-221.

38. Donaldson DR, Salter MCP, Kester RC, Rajah SM, Hall TJ, et al. (1985) The influence of platelet inhibition on the patency of femoro-popliteal Dacron bypass grafts. Vasc Surg 19: 224-230.

39. Hess H, Mietaschk A, Deichsel G (1985) Drug-induced inhibition of platelet function delays progression of peripheral occlusive arterial disease. A prospective double-blind arteriographically controlled trial. Lancet 1: 415-419.

40. Colwell JA, Bingham SF, Abraira C, Anderson JW, Comstock JP, et al. (1986) Veterans Administration Cooperative Study on antiplatelet agents in diabetic patients after amputation for gangrene: II. Effects of aspirin and dipyridamole on atherosclerotic vascular disease rates. Diabetes Care 9: 140-148.

41. Heiss HW, Just H, Middleton D, Deichsel G (1990) Reocclusion prophylaxis with dipyridamole combined with acetylsalicylic acid following PTA. Angiology 41: 263-269.

42. McCollum C, Alexander C, Kenchington G, Franks PJ, Greenhalgh R (1991) Antiplatelet drugs in femoropopliteal vein bypasses: a multicenter trial. J Vasc Surg 13: 150-161; discussion 161-152.

43. Catalano M, Born G, Peto R (2007) Prevention of serious vascular events by aspirin amongst patients with peripheral arterial disease: randomized, double-blind trial. J Intern Med 261: 276-284.

44. Aukland A, Hurlow RA, George AJ, Stuart J (1982) Platelet inhibition with Ticlopidine in atherosclerotic intermittent claudication. J Clin Pathol 35: 740-743.

45. Stiegler H, Hess H, Mietaschk A, Trampisch HJ, Ingrisch H (1984) Einfluß von ticlopidin auf die periphere obliterierende arteriopathie. . Dtsch Med Wochenschr 109: 1240-1243.

46. Castelli P, Basellini A, Agus GB, Ippolito E, Pogliani EM, et al. (1986) Thrombosis prevention with ticlopidine after femoropopliteal thromboendarterectomy. Int Surg 71: 252-255.

47. Ellis DJ (1986) Treatment of intermittent claudication with ticlopidine. . Proceedings of International Committee on Thrombosis and Haemostasis 32nd Meeting in Jerusalem: 60:60 (Abstract addendum)

48. Arcan JC, Blanchard J, Boissel JP, Destors JM, Panak E (1988) Multicenter double-blind study of ticlopidine in the treatment of intermittent claudication and the prevention of its complications. Angiology 39: 802-811.

49. Balsano F, Coccheri S, Libretti A, Nenci GG, Catalano M, et al. (1989) Ticlopidine in the treatment of intermittent claudication: a 21-month double-blind trial. J Lab Clin Med 114: 84-91.

50. Bergqvist D, Almgren B, Dickinson JP (1995) Reduction of requirement for leg vascular surgery during long-term treatment of claudicant patients with ticlopidine: results from the Swedish Ticlopidine Multicentre Study (STIMS). Eur J Vasc Endovasc Surg 10: 69-76.

51. Janzon L, Bergqvist D, Boberg J, Boberg M, Eriksson I, et al. (1990) Prevention of myocardial infarction and stroke in patients with intermittent claudication; effects of ticlopidine. Results from STIMS, the Swedish Ticlopidine Multicentre Study. J Intern Med 227: 301-308.

52. Blanchard J, Carreras LO, Kindermans M (1994) Results of EMATAP: a double-blind placebo-controlled multicentre trial of ticlopidine in patients with peripheral arterial disease. Nouv Rev Fr Hematol 35: 523-528.

53. Blanchard JF, Carreras LO (1992) A double-blind, placebo-controlled multicentre trial of ticlopidine in patients with peripheral arterial disease in Argentina. Design, organization and general characteristics of patients at entry. The EMATAP Group. Nouv Rev Fr Hematol 34: 149-153.

54. (1996) A randomised, blinded, trial of clopidogrel versus aspirin in patients at risk of ischaemic events (CAPRIE). CAPRIE Steering Committee. Lancet 348: 1329-1339.

55. Steinhubl SR (2006) Clopidogrel bisulfate: in ST-segment elevation myocardial infarction: a viewpoint by Steven R Steinhubl. Am J Cardiovasc Drugs 6: 415-416.

56. Mukherjee D, Topol EJ, Moliterno DJ, Brennan DM, Ziada K, et al. (2006) Extracardiac vascular disease and effectiveness of sustained clopidogrel treatment. Heart 92: 49-51.

57. Bhatt DL, Flather MD, Hacke W, Berger PB, Black HR, et al. (2007) Patients with prior myocardial infarction, stroke, or symptomatic peripheral arterial disease in the CHARISMA trial. J Am Coll Cardiol 49: 1982-1988.

58. Cacoub PP, Bhatt DL, Steg PG, Topol EJ, Creager MA (2009) Patients with peripheral arterial disease in the CHARISMA trial. Eur Heart J 30: 192-201.

59. Belch JJ, Dormandy J, Biasi GM, Cairols M, Diehm C, et al. (2010) Results of the randomized, placebo-controlled clopidogrel and acetylsalicylic acid in bypass surgery for peripheral arterial disease (CASPAR) trial. J Vasc Surg 52: 825-833, 833 e821-822.

60. Tepe G, Bantleon R, Brechtel K, Schmehl J, Zeller T, et al. (2012) Management of peripheral arterial interventions with mono or dual antiplatelet therapy--the MIRROR study: a randomised and double-blinded clinical trial. Eur Radiol 22: 1998-2006.

61. Strobl FF, Brechtel K, Schmehl J, Zeller T, Reiser MF, et al. (2013) Twelve-month results of a randomized trial comparing mono with dual antiplatelet therapy in endovascularly treated patients with peripheral artery disease. J Endovasc Ther 20: 699-706.

62. Wallentin L, Becker RC, Budaj A, Cannon CP, Emanuelsson H, et al. (2009) Ticagrelor versus clopidogrel in patients with acute coronary syndromes. N Engl J Med 361: 1045-1057.

63. Serebruany VL (2012) Peripheral vascular outcomes in the PLATO trial: update from the FDA ticagrelor complete response review. Am J Ther 19: 160-161.

64. Patel MR, Becker RC, Wojdyla DM, Emanuelsson H, Hiatt WR, et al. (2011) Cardiovascular Events in Acute Coronary Syndrome Patients With Peripheral Arterial Disease Treated With Ticagrelor Compared to Clopidogrel: Data From the PLATO Trial. Circulation 124: A14299.

65. Patel MR, Becker RC, Wojdyla DM, Emanuelsson H, Hiatt WR, et al. (2014) Cardiovascular events in acute coronary syndrome patients with peripheral arterial disease treated with ticagrelor compared with clopidogrel: Data from the PLATO Trial. Eur J Prev Cardiol.

66. Shigematsu H, Komori K, Tanemoto K, Harada Y, Nakamura M (2012) Clopidogrel for Atherothrombotic Event Management in Patients with Peripheral Arterial Disease (COOPER) Study: Safety and Efficacy of Clopidogrel versus Ticlopidine in Japanese Patients. Ann Vasc Dis 5: 364-375.

67. Beebe HG, Dawson DL, Cutler BS, Herd JA, Strandness DE, Jr., et al. (1999) A new pharmacological treatment for intermittent claudication: results of a randomized, multicenter trial. Arch Intern Med 159: 2041-2050.

68. Strandness DE, Jr., Dalman RL, Panian S, Rendell MS, Comp PC, et al. (2002) Effect of cilostazol in patients with intermittent claudication: a randomized, double-blind, placebo-controlled study. Vasc Endovascular Surg 36: 83-91.

69. Katakami N, Kim YS, Kawamori R, Yamasaki Y (2010) The phosphodiesterase inhibitor cilostazol induces regression of carotid atherosclerosis in subjects with type 2 diabetes mellitus: principal results of the Diabetic Atherosclerosis Prevention by Cilostazol (DAPC) study: a randomized trial. Circulation 121: 2584-2591.

70. Hiatt WR, Money SR, Brass EP (2008) Long-term safety of cilostazol in patients with peripheral artery disease: the CASTLE study (Cilostazol: A Study in Long-term Effects). J Vasc Surg 47: 330-336.

71. Soga Y, Yokoi H, Kawasaki T, Nakashima H, Tsurugida M, et al. (2009) Efficacy of cilostazol after endovascular therapy for femoropopliteal artery disease in patients with intermittent claudication. J Am Coll Cardiol 53: 48-53.

72. Iida O, Yokoi H, Soga Y, Inoue N, Suzuki K, et al. (2013) Cilostazol reduces angiographic restenosis after endovascular therapy for femoropopliteal lesions in the Sufficient Treatment of Peripheral Intervention by Cilostazol study. Circulation 127: 2307-2315.

73. Bonaca MP, Scirica BM, Creager MA, Olin J, Bounameaux H, et al. (2013) Vorapaxar in patients with peripheral artery disease: results from TRA2{degrees}P-TIMI 50. Circulation 127: 1522-1529, 1529e1521-1526.

74. Coto V, Cocozza M, Oliviero U, Lucariello A, Picano T, et al. (1989) Clinical efficacy of picotamide in long-term treatment of intermittent claudication. Angiology 40: 880-885.

75. Balsano F, Violi F (1993) Effect of picotamide on the clinical progression of peripheral vascular disease. A double-blind placebo-controlled study. The ADEP Group. Circulation 87: 1563-1569.

76. Neirotti M, Molaschi M, Ponzetto M, Macchione C, Poli L, et al. (1994) Hemodynamic, hemorheologic, and hemocoagulative changes after treatment with picotamide in patients affected by peripheral arterial disease (PAD) of the lower limbs. Angiology 45: 137-141.

77. Neri Serneri GG, Coccheri S, Marubini E, Violi F (2004) Picotamide, a combined inhibitor of thromboxane A2 synthase and receptor, reduces 2-year mortality in diabetics with peripheral arterial disease: the DAVID study. Eur Heart J 25: 1845-1852.
